# Histological and mutational profile of diffuse gastric cancer: current knowledge and future challenges

**DOI:** 10.1002/1878-0261.12948

**Published:** 2021-05-02

**Authors:** José Garcia‐Pelaez, Rita Barbosa‐Matos, Irene Gullo, Fátima Carneiro, Carla Oliveira

**Affiliations:** ^1^ i3S – Instituto de Investigação e Inovação em Saúde da Universidade do Porto Portugal; ^2^ IPATIMUP – Institute of Molecular Pathology and Immunology University of Porto Portugal; ^3^ Doctoral Programme on Biomedicine Faculty of Medicine University of Porto Portugal; ^4^ Doctoral Programme on Cellular and Molecular Biotechnology Applied to Health Sciences (BiotechHealth) ICBAS – Institute of Biomedical Sciences Abel Salazar University of Porto Portugal; ^5^ Department of Pathology FMUP ‐ Faculty of Medicine of the University of Porto Portugal; ^6^ Department of Pathology CHUSJ – Centro Hospitalar Universitário São João Porto Portugal

**Keywords:** diffuse gastric cancer, driver genes, intestinal gastric cancer, molecular classification, poorly cohesive carcinomas, signet‐ring cell carcinoma

## Abstract

Gastric cancer (GC) pathogenesis is complex and heterogeneous, reflecting morphological, molecular and genetic diversity. Diffuse gastric cancer (DGC) and intestinal gastric cancer (IGC) are the major histological types. GC may be sporadic or hereditary; sporadic GC is related to environmental and genetic low‐risk factors and hereditary GC is caused by inherited high‐risk mutations, so far identified only for the diffuse histotype. DGC phenotypic heterogeneity challenges the current understanding of molecular mechanisms underlying carcinogenesis. The definition of a DGC‐specific mutational profile remains controversial, possibly reflecting the heterogeneity of DGC‐related histological subtypes [signet‐ring cell carcinoma (SRCC) and poorly cohesive carcinoma not otherwise specified (PCC‐NOS)]. Indeed, DGC and DGC‐related subtypes may present specific mutational profiles underlying the particularly aggressive behaviour and dismal prognosis of DGC vs IGC and PCC‐NOS vs SRCC. In this systematic review, we revised the histological presentations, molecular classifications and approved therapies for gastric cancer, with a focus on DGC. We then analysed results from the most relevant studies, reporting mutational analysis data specifying mutational frequencies, and their relationship with DGC and IGC histological types, and with specific DGC subtypes (SRCC and PCC‐NOS). We aimed at identifying histology‐associated mutational profiles with an emphasis in DGC and its subtypes (DGC vs IGC; sporadic vs hereditary DGC; and SRCC vs PCC‐NOS). We further used these mutational profiles to identify the most commonly affected molecular pathways and biological functions, and explored the clinical trials directed specifically to patients with DGC. This systematic analysis is expected to expose a DGC‐specific molecular profile and shed light into potential targets for therapeutic intervention, which are currently missing.

## Graphical abstract

The main diffuse gastric cancer (DGC) histological subtypes are signet‐ring cell carcinoma (SRCC) and poorly cohesive carcinoma not otherwise specified (PCC‐NOS). Clinical trials, targeted therapies and predictive markers of therapy response directed to any of these entities are residual. This systematic review exposes the lack of integrated knowledge in these clinical entities and uses their mutational profiles to rethink therapeutic intervention.

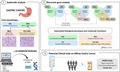

**Abbreviations**
DGC: diffuse gastric cancer
IGC: intestinal gastric cancer
PCC‐NOS: poorly cohesive carcinoma not otherwise specified
SRCC: signet‐ring cell carcinoma

## 1 Introduction – a general overview of gastric cancer morphology and mutational landscape

Gastric cancer (GC) is very heterogeneous from the morphological, genetic and molecular standpoints. The molecular heterogeneity is mirrored in the great variability of morphological phenotypes [1]. In this work, we revised the histological features, molecular classifications and approved therapies for GC, with a focus on DGC. We then explored a set of studies that reported mutational analysis data specifying mutational frequencies and their relationship with DGC and IGC histological types, as well as with specific subtypes of DGC, namely SRCC and poorly cohesive carcinoma not otherwise specified (PCC‐NOS), to fill a gap in the current knowledge.

### 1.1 Main histopathological classifications of gastric cancer

A large number of histopathological classifications have been proposed over time [2, 3, 4, 5, 6, 7, 8]. The most commonly used gastric cancer classification is, in the Western countries, the one proposed by Laurén [3] and the World Health Organization (WHO) classification [8]. In Eastern countries, the most commonly used gastric cancer classification is the one issued by the Japanese Gastric Cancer Association (JGCA) [7], which is very similar to the WHO classification, 5th edition [9].

The Laurén classification [3] distinguishes two major types, intestinal gastric cancer (IGC) and diffuse gastric cancer (DGC). The former is constituted by tubular or papillary structures, while the latter is characterized by poorly cohesive and infiltrative tumour cells that may or may not have a signet‐ring cell (SRC) morphology. Tumours presenting both intestinal and diffuse components are termed mixed carcinomas. Other carcinomas that do not fit in one of these subtypes are placed in the indeterminate category. Despite dating back to 1965, Laurén classification is still widely accepted and used, as it distinguishes subtypes with distinct epidemiologic settings, clinicopathologic profiles and biological behaviours. These subtypes also correspond to distinct tumour spreading patterns: while IGC tends to metastasize haematogenously to the liver, DGC usually disseminates through peritoneal surfaces. Mixed gastric cancer shows a poorer prognosis compared with IGC or DGC types [10, 11] and a dual metastatic pattern (haematogenous metastases and peritoneal dissemination with lymph node metastases) [12], probably because of the cumulative adverse effect of the two components within a single tumour.

The classification of the World Health Organization (WHO), 5th edition (2019), [9] recognizes five major types of gastric adenocarcinoma (tubular, papillary, mucinous, poorly cohesive and mixed), as well as other rarer subtypes (gastric carcinoma with lymphoid stroma, hepatoid adenocarcinoma and related entities, micropapillary adenocarcinoma, gastric adenocarcinoma of fundic gland type, etc.).

### 1.2 The morphological heterogeneity of DGC

The DGC subtype in Laurén classification roughly corresponds to the poorly cohesive carcinomas (PCC) in the 2019 WHO classification [8].

**Fig. 1:**
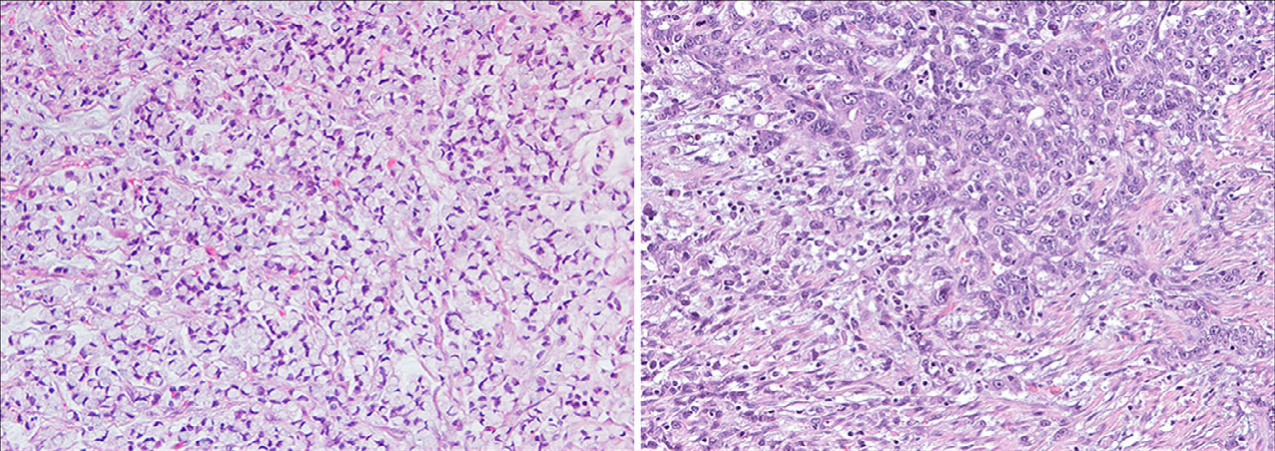
Representative Images of a SRCC case (left) and a PCC‐NOS case (right) highlighting the heterogeneity present in sporadic DGC.

DGC/PCC may show great morphological variability, between different tumours from distinct patients, but also within different areas of the same tumour (intertumour and intratumour morphological heterogeneity). The classical picture of DGC/PCC displays a tumour composed of poorly cohesive signet‐ring cells (SRCs) (Fig. 1, left panel). SRCs are defined by the presence of an abundant mucin vacuole filling the cytoplasm and pushing the nucleus to the cell periphery [13]. Especially in early lesions (i.e., GCs limited to the mucosa and submucosa), different types of SRCs may be observed, which are distinguished by the nature of the cytoplasmic mucin [14, 15]. The description of different SRC types may be considered merely academic, as it does not appear to influence the tumour prognosis. DGC/PCC may also show cells that do not present classic SRC morphology. In particular, advanced lesions infiltrating the gastric wall may show pleomorphic, bizarre and diffusely infiltrative neoplastic cells, sometimes with a lymphohistiocytic or sarcomatoid appearance, the latter representing the surrogate of epithelial‐to‐mesenchymal transition activation. The recognition of this subtype is of utmost importance, as it presents a poorer prognosis when compared to pure SRC carcinomas (SRCCs) [16]. Consistent with this observation, the new 2019 WHO classification stresses the importance of evaluating the morphological heterogeneity of DGC and distinguishing different PCC subtypes, based on the presence and quantity of SRCs [8]. Poorly cohesive carcinomas of the SRC type (SRCC) are composed predominantly or exclusively of SRCs and show a better prognosis, while non‐SRC type, that is PCC‐NOS, is composed (or show a predominant component) of poorly cohesive and cells, without a classic SRC morphology (Fig. 1, right panel).

### 1.3 Main molecular classifications of gastric cancer

The molecular and genomic analysis of large GC series has identified a large variety of oncogene activating mutations, tumour suppressor gene (TSG)‐inactivating alterations, gene fusions, somatic copy number alterations (sCNAs) and other structural variations, as well as epigenetic and transcriptional changes [17]. Activating mutations in oncogenes such as *KRAS*, *BRAF* and *PIK3CA* have been reported in GC through single‐gene analysis at least three decades ago [18, 19, 20, 21] and remain important in GC using genome‐wide strategies [22, 23, 24]. *TP53* is the most frequently mutated TSG in GC, often occurring in more than 50% of the cases, regardless of the experimental approach used, either target gene or genome‐wide [22, 25, 26, 27]. Somatic inactivating mutations in *CDH1* gene (a relevant TSG in GC) are known since the early 90’s and remain a hallmark of the diffuse histological type [22, 28, 29, 30, 31, 32, 33, 34].

Genome‐wide strategies allowed to overcome the limitations of single‐gene targeted approaches. Through next‐generation sequencing (NGS) technologies, the knowledge on GC mutational burden and molecular signatures increased which culminated in the development of GC molecular classifications. These classifications based on genomics, transcriptomics and/or epigenomic profiles can be associated with the main GC histotypes (Table 1).

**Table 1:** Overview of gastric cancer molecular classifications and relationship with main Laurén histotypes. ACRG, Asian Cancer Research Group; CIMP, CpG island methylation phenotype; CIN, chromosomal instability; CNA, copy number alteration; CSC, cancer stem cell; EBV, Epstein–Barr virus; EMT, epithelial‐to‐mesenchymal transition; GC, gastric cancer; GS, genomically stable; MSI, microsatellite instability; MSS, microsatellite stable; TCGA, The Cancer Genome Atlas; TKR, tyrosine kinase receptors. Lauren histological subtypes are highlighted in green (diffuse GC) and light blue (intestinal GC).

Reference (sample size)	Methodology	Main features of molecular subtypes and relationship with Laurén histopathological classification				
[35] (*n* = 270)	Gene expression profiling			**G‐DIF** (44%)	**G‐INT** (56%)	
				Cell proliferation Fatty acid metabolism 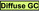	Cell adhesion Carbohydrate and protein metabolism 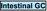	
[36] (*n* = 248)	Gene expression profiling, *TP53* mutation, CNA and ;DNA methylation analysis			**MESENCHYMAL**	**PROLIFERATIVE**	**METABOLIC**
				Low *TP53* mutations Low E‐cadherin mRNA CSC/EMT proprieties 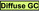	High *TP53* mutations Genomic instability Oncogene amplification 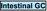 (intestinal phenotype)	Low *TP53* mutations Normal gastric mucosa gene expression 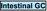 (gastric phenotype)
[22] (*n* = 295) – TCGA cohort	Array‐based somatic copy number analysis, whole‐exome seq, array‐based DNA methylation profiling, mRNA seq, microRNA seq and reverse‐phase protein array, MSI testing.	**EBV** (9%)	**MSI** (22%)	**GS** (20%)	**CIN** (50%)	
		EBV‐CIMP *CDKN2A* silencing *PIK3A* mutations *PD‐L1/2* amplification *JAK2* amplification	Gastric‐CIMP *MLH1* silencing *PIK3A* mutations *HER2/3* mutations *EGFR* mutations 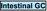	*CDH1* mutations *RHOA* mutations *CLDN18*‐*ARHGAP* fusion (RhoA‐GTPase) 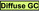	High *TP53* mutations *TKR‐RAS* amplification Amplification of cell‐cycle mediators 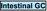	
[37] (*n* = 251) – ACRG cohort	Whole‐genome seq, gene expression profiling, copy number analysis, targeted re‐sequencing.		**MSI** (23%)	**MSS/EMT** (15%)	**MSS/TP53‐** (36%)	**MSS/TP53+ (**26%)
		EBV+ cases included in MSS/TP53+	*MLH1* loss Hypermutation (KRAS, ARID1A, PIK3A) 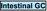	*CDH1* loss 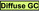	High *TP53* mutations Genomic instability Oncogene amplification 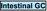	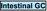

The first GC molecular classification was based exclusively on gene expression profiling and directly addressed differences in GC main histological types [35]. The second, although driven by gene expression profiling, was supported by gene‐specific mutational analysis, chromosomal instability and DNA methylation analysis [36]. These studies also presented patients’ response to treatment and disease outcomes according to molecular classification. In 2014, a game‐changer GC molecular classification was published by The Cancer Genome Atlas (TCGA) consortium [22], based on a deep and integrative molecular profiling with array‐based somatic copy number analysis, whole‐exome sequencing, array‐based DNA methylation profiling, messenger RNA sequencing (mRNA), microRNA (miRNA) sequencing and reverse‐phase protein array (RPPA), microsatellite instability (MSI) testing and low‐pass whole‐genome sequencing. In 2015, the Asian Cancer Research Group (ACRG) generated comprehensive genomic and transcriptomic data sets from 300 GC cases, mainly from East Asia, producing the first GC classification based on integrated molecular data analysis and clinical outcomes [37]. In the two latter publications, there was an attempt to correlate the molecular subtypes with GC main histotypes, which did not provide a straightforward overlap (Table 1). Nevertheless, there is some correspondence between the two latter classifications, namely regarding tumours bearing mismatch repair deficiency (MSI), and those dominated by E‐cadherin deficiency, genomic stability and epithelial‐to‐mesenchymal transition features (GS for TCGA and MSS/EMT for ACRG). The greater differences between TCGA and ACRG classifications relate to the lack of an EBV subgroup in the ACRG classification, which is molecularly defined in the TCGA study, and the heterogeneity of the CIN subgroup in TCGA, which becomes divided into two distinct subgroups in the ACRG study (Table 1).

### 1.4 Cost‐effective strategies for molecular classification

**Fig. 2:**
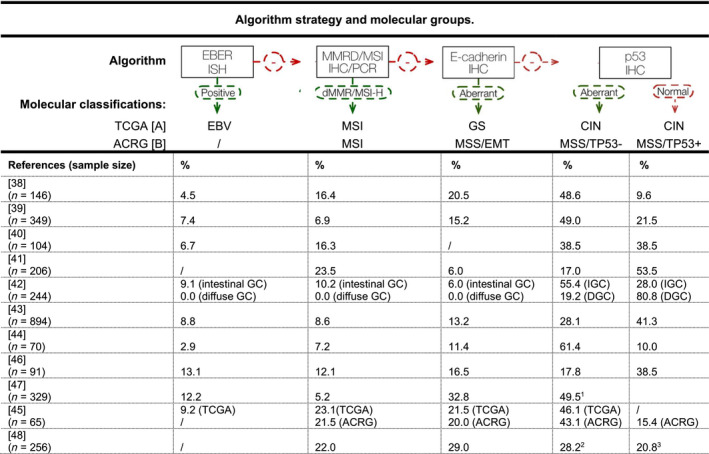
Cost‐effective strategies for molecular classification. [A] TCGA, The Cancer Genome Atlas [22]; [B] ACRG, Asian Cancer Research Group [37]; CIN, chromosomal instability; EBER ISH, EBV‐encoded small RNA *in situ* hybridization; EBV, Epstein–Barr virus; EMT, epithelial‐to‐mesenchymal transition; GC, gastric cancer; GS, genomically stable; IHC, immunohistochemistry; MMRD, mismatch repair deficiency; MSI, microsatellite instability; MSS, microsatellite stability; PCR, polymerase chain reaction. ^1^CIN subtype defined by the presence of aneuploidy, using DNA flow cytometry; ^2^MSS/TP53‐ subtype defined by low p21 protein expression; ^3^MSS/TP53+ subtype defined by high p21 protein expression.

Given the complexity and high cost of the experimental approaches needed to stratify GC according to TCGA and ACRG classifications, several studies proposed less costly approaches to reach similar endpoints (Fig. 2) [38, 39, 40, 41, 42, 43, 44, 45, 46, 47, 48].

In 2016, Setia *et al*. used 14 biomarkers in a GC cohort from the United States and Ahn *et al*. studied protein or mRNA expression of MLH1, E‐cadherin, p53 and EBV in a South Korean population to derive five GC molecular subgroups and reproducing a combination of TCGA and ACRG classifications [38, 39]. In 2016, *in situ* hybridization (ISH) or immunohistochemistry (IHC) were used to study MLH1, p53 and EBV, but excluded the analysis of MSS/EMT (ACRG) or GS (TCGA) [40]. In 2018, GC patients were classified using IHC based on molecular biomarkers to derive four molecular subgroups, which excluded EBV, and supported this classification demonstrating a significant and independent correlation between IHC subtype and overall survival, clinical outcome, SRC phenotype, tumour grade and vessel invasion [41].

In parallel, in 2018, ISH and IHC were performed in a cohort mainly composed of IGC to refine previous GC classifications [42]. In 2019, 894 consecutive GC Korean patients were used to validate previous classifications, with IHC‐ and PCR‐based approaches and confirming the association between molecular subtypes and patients’ overall survival [43]. More recently, in 2020, an immunophenotypic classification, based on IHC and ISH, was used to evaluate MLH1, p53, HER2, E‐cadherin and EBV in whole sections from surgical GC specimens [44]. Another similar approach was used to apply the IHC/ISH molecular algorithm to a cohort of GC patients from Chile [46]. In this study, the authors used targeted NGS sequencing to characterize the four molecular subtypes, identifying *FGFR2* and *KRAS* gene amplification as potential actionable targets in the EMT‐like subgroup [46]. Wang *et al*. [45] studied a GC Chinese cohort to conclude that it would be possible to reproduce the TCGA and ACRG molecular classifications only by using IHC. In the two most recent cost‐effective strategies for GC molecular classification, Zhao *et al*. and Tsai *et al*. used alternative surrogate biomarkers to define MSS/TP53 subgroups and CIN subtype, based on p21 protein expression and DNA content assessed by DNA flow cytometry, respectively [47, 48].

### 1.5 Clinical outcomes, molecular features, prognosis, and approved and promising therapies for GC molecular subgroups

The research effort that followed the publication of the TCGA and ACRG molecular classifications led not only to the development of cost‐effective strategies to stratify GC patients according to these classifications (Fig. 2), but also to validate the molecular components from different molecular subgroups. This further allowed correlating molecular subgroups or alterations with clinical outcomes and response to therapy (Table 2). In Table 2, the possible correlations between TCGA and ACRG subgroups are presented, supported by shared molecular features. This information allows rethinking the application of former approved therapies and novel promising ones.

**Table 2:** Representative genomic subtypes, common molecular features, clinical outcomes and corresponding therapeutic approaches. NA, not available; The Cancer Genome Atlas (TCGA); Asian Cancer Research Group (ACRG); Genomically stable (GS); Microsatellite Stable/Epithelial‐Mesenchymal transition (MSS/EMT); Microsatellite instable (MSI; Helicobacter pylori (H. pylori); Vascular endothelial growth factor receptor 2 tyrosine kinase (VEGFR2); Programmed‐cell‐death‐1‐receptor (PD‐1); Programmed death‐ligand 1 (PD‐L1); Receptor Tyrosine Kinase (RTK); Human epidermal growth factor receptor 2 (HER2); Epidermal growth factor receptor(EGFR); Fibroblast Growth Factor Receptor 2 (FGFR2); Poly (ADP‐ribose) polymerase (PARP); ataxia telangiectasia and Rad‐3 related protein kinase (ATR); References: [A] [22]; [B] [37] [C] [49] [D] [83]; [E] [84]; [F] [85]; [G] [86]; [H] [87] (e‐updated in 2019); [I] [52]; [J] [88]; [K] [89]; [L] [53]; [M] [90]; [N] [91]; [O] [54]; [O] [92]; [P] [57]; [Q] [93]; [R] [56].

TCGA molecular classification [A]			ACRG molecular classification [B]			Shared features and therapeutic approach		
Subtype frequency main location predominant histology	Clinical outcome/ prognosis	Molecular features	Subtype frequency main location predominant histology	Clinical outcome/ prognosis	Molecular features	Shared molecular features	Approved therapeutic strategies	Promising targets/strategies
GS			MSS/EMT			GS and MSS/EMT		
20% NA DGC	Diagnosed at early age. Worst overall survival. Less benefit from adjuvant chemotherapy [C]. Poor response to immune checkpoint inhibitors [D][E][F].	High frequency of *CDH1* (37%) and *RHOA* (15%) mutations. *CLDN18*‐*ARHGAP* fusion. Elevated expression of angiogenesis‐related pathways [A].	15% NA DGC	Poor prognosis and high recurrence frequency with tendency to develop at earlier age. Majority of cases diagnosed at stages III/IV [B].	Loss of E‐cadherin expression. Low mutation rate [B].	*CDH1* and *RHOA* mutations	Standard chemotherapy: combinations of fluoropyrimidines and platinum derivatives [G][H].	Cell junctions: CLDN18.2 antibody (phase III clinical trials: NCT03504397, NCT03653507); intraperitoneal administration of anticancer agents (phase III clinical trial: UMIN000005930 [I]; (phase II clinical trial NCT04034251); anti‐VEGFR2 treatment using ramucirumab and apatinib [J]; [K] and other angiogenesis inhibitors (phase III clinical trial NCT02773524) [L][M]
MSI			MSI			MSI		
22% antrum IGC	Diagnosed at older ages; associated with *H. pylori* infection and intestinal metaplasia [A]. Better prognosis than the MSS subtype [C]	CpG island methylator phenotype (*MLH1* silencing): hypermutation phenotype frequently targeting genes such as *TP53*, *KRAS*, *ARID1A*, *PIK3CA*, *ERBB3*, *PTEN*, *CHRD* and *HLA‐B* with nucleotide substitutions; and RNF43, B2 M and NF1 with insertions/deletions [A][N].	23% antrum IGC	Good prognosis [B].	*MLH1* gene silencing; commonly mutated genes: *ARID1A*, *MTOR*,*KRAS*, *PIK3CA*, *ALK*, *PTEN*. Overexpression of PD‐L1; T‐cell infiltration [B].	Hypermutated and hypermethylated tumours. Both enriched in IGCs.	Immune checkpoint inhibitors: Anti‐PD‐1 [L][O][P][R].	Immune checkpoint inhibitors: PD‐1/PD‐L1 pathway: (phase III clinical trials: NCT02872116, NCT02746796, NCT03019588, NCT03615326, NCT03189719, NCT02625610, NCT04152889) [L][M].
EBV +			MSS/TP53+			EBV+ and MSS/TP53+		
9% Gastric fundus or body Gastric cancer with lymphoid stroma	Good prognosis [C].	EBV infection; CpG island methylation; *CCDKN2A* silencing; *JAK2*, *CD274*, *PDCD1LG2,* *ERBB2, PD‐L1 and PD‐L2* amplification; mutations in *PIK3CA* (80%), *ARID1A* (55%) and *BCOR* (23%) [A].	26% NA IGC	Intermediate prognosis [B].	Mutations in *ARID1A*, *PIK3CA*, *SMAD4*, *APC* [B].	EBV infection; *PIK3CA* mutations	Standard chemotherapy: combinations of fluoropyrimidines and platinum derivatives [G][H].	Immune checkpoint inhibitors: PD‐1/PD‐L1 pathway: (phase III clinical trials NCT02872116, NCT02746796, NCT03019588, NCT03615326, NCT03189719, NCT02625610, NCT04152889) and (phase II clinical trials NCT03755440, NCT03257163 and NCT04202601) [L][M][P][Q][R].
CIN			MSS/TP53‐			CIN and MSS/TP53‐		
50% Gastro‐oesophageal junction/cardia IGC	Poorer overall survival than EBV subtype but better than GS. Benefit from adjuvant chemotherapy [C]. Poor response to immune checkpoint inhibitors [E][F].	RTK‐RAS pathway genes amplification namely *HER2*, *VEGFA* and *FGFR2*; *TP53* (71%) mutations [A][O].	36% NA IGC	Intermediate prognosis and recurrence rates compared with the other subtypes. Survival benefit with adjuvant chemotherapy [B].	Genomic instability; *TP53* mutations; recurrent focal amplification of RTKs and cell cycle modulators; *ERBB2* (HER2) amplification[B].	High frequency of *TP53* mutations. Alterations in RTK‐RAS pathway genes. Both enriched in IGCs	Standard chemotherapy: Combinations of fluoropyrimidines and platinum derivatives [G][H]. Trastuzumab for HER2‐positive GCs [L][P].	RTK‐RAS pathway: EGFR, HER2 and HER4 inhibition (phase II/III clinical trial NCT03130790); EGFR overexpression ( phase III clinical trial NCT01813253); FGFR2 amplification (phase III clinical trial NCT0369452 and phase II clinical trial NCT03694522); anti‐VEGFR2 treatment using ramucirumab and apatinib [J][K], and other angiogenesis inhibitors (phase III clinical trial NCT02773524); DNA repair: PARP inhibitors (phase II clinical trials NCT03427814, NCT02678182) TP53 mutants: ATR inhibitor (phase II clinical trial NCT03641313) [L][M].

One of the most evident gaps in GC therapy is the lack of targeted therapies for DGC patients, which mainly reside in the TCGA GS group and in the MSS/EMT ACRG group (Table 2). This group of patients, which represents nearly 20% of all GC and those with the poorest prognosis, is currently treated with conventional chemotherapy but responds poorly [49] (Table 2). Immune checkpoint inhibitors did not prove as an alternative in these patients, as MSS/EMT cases from the ACRG group also showed poor treatment response [37]. Promising alternative therapies such as anti‐CLDN18.2 antibody, targeting the *CLDN18‐ARHGAP26* gene fusion, which is enriched in DGC cases [22, 50, 51] and intraperitoneal administration of anticancer agents [52], are just emerging treatments for this molecular subgroup, but results are still pending (Table 2).

The remaining GC molecular subgroups are less problematic regarding prognosis and available approved therapies or emerging ones (Table 2). The subgroups EBV‐positive from TCGA and MSI from TCGA and ACRG show the best prognosis and are more likely to benefit from immune checkpoints inhibitors. In fact, MSI tumours are already selected for anti‐PD1 treatment [53, 54, 55]. Patients bearing EBV‐positive tumours are part of the ACRG MSS/TP53+ subgroup, present intermediate prognosis and are also often PD‐L1‐positive, turning them also into good candidates for immune checkpoint inhibition. Indeed, a phase II study on pembrolizumab showed the ORR to be not only 85.7% in MSI‐H gastric cancers, but also more important to be 100% in EBV‐related GC [56]. The TCGA CIN and ACRG MSS/TP53‐ subgroups present intermediate prognosis and the best survival benefit from adjuvant chemotherapy [37]. Given the high frequency of oncogene amplifications, tumours from this subgroup may also be treated with the approved anti‐HER2 therapy [57], and promising treatments include inhibitors of the RTK‐RAS pathway, DNA repair molecules and ATR (Table 2).

Diffuse gastric cancer has long been recognized as an independent GC histopathological entity; however, along the time, DGC‐associated subtypes have been recognized and deeply characterized at the morphological and molecular levels. Despite this recognition, the most two comprehensive GC molecular classifications (TCGA and ACRG) have been blind to these subtypes, and DGC as a whole, mainly cluster in the TCGA GS group and in the MSS/EMT ACRG group. These two molecular subgroups, besides sharing a dominant GC histotype, also share mutations in DGC‐associated genes. Despite the increasing knowledge about GC histopathological and molecular subtypes, the integration of these two aspects and its clinical utility remains poorly explored.

In summary, DGC and its subtypes remain a major problem in terms of prognosis, response to conventional chemotherapy and a still arid ground for novel therapeutic interventions.

### 1.6 Aim

After recognizing a knowledge gap regarding molecular features potentially useful for clinical outcome prediction and therapy design in DGC, our aim was to identify histology‐associated mutational profiles for this GC histotype and its subtypes. We further used these mutational profiles to identify their most affected molecular pathways and biological functions, and explored the clinical trials specifically designed for DGC patients. This systematic analysis is expected to expose a DGC‐specific molecular profile and shed light into potential targets for therapeutic intervention, which are currently missing.

## 2 Methodology

We collected available literature from 1998 to 2020, in order to get an overall view of the morphological and molecular features of sporadic GC and its molecular classifications. Peer‐reviewed published articles were searched with the help of ‘PubMed’ database using the following keywords in different combinations: ‘mutational landscape, mutational signature, gastric cancer, diffuse‐type gastric cancer, poorly cohesive carcinoma and signet‐ring cell carcinoma’. Only peer‐reviewed published articles written in English language, irrespective of their study design and type (case reports, case series, original research articles, reviews, systematic reviews, meta‐analyses, cohort studies, clinical trials), were consulted, and in the context of the present study, we selected only articles with relevant data and best fitting our aim.

### 2.1 Data collection for recurrent gene analysis

For the recurrent gene analysis, published studies or public cohorts reporting mutational frequencies and their relationship with GC histological types and subtypes of interest (IGC, DGC, SRCC and PCC‐NOS) were searched for, selected upon compliance with the following criteria and refined by manual curation:

1. Studies from CBIOPORTAL (https://www.cbioportal.org/) encompassing ‘gastric/ stomach adenocarcinoma’ cancer NGS data;
2. ‘PubMed’ deposited peer‐reviewed articles, published between 2014 and 2020, reporting on data produced by targeted sequencing and/or whole‐exome sequencing and/or whole‐genome sequencing, and according to different MESH term combinations:
  - (mutational landscape OR mutational signature) AND (diffuse‐type gastric carcinoma OR diffuse‐type gastric cancer OR gastric adenocarcinoma OR gastric cancer OR poorly cohesive carcinoma) NOT review.
  - (genomic variation) AND (diffuse‐type gastric cancer) NOT review.
  - (target sequencing OR whole‐exome sequencing OR whole‐genome sequencing) AND (diffuse‐type gastric cancer) NOT review.

Manual curation was further performed on all publications selected with the above criteria, aiming at finding those presenting histological classification for each tumour, either according to Laurén [3] for comparison between DGC and IGC, or specifically mentioning comparisons between DGC subtypes, and not restricted to Laurén classification, namely if mentioning SRCC and PCC‐NOS. Moreover, to be selected for the analysis, each publication should also specify either the set of mutated genes per case, or the gene mutation frequency among histological types or subtypes, as well as the total number of cases analysed within the materials (main or supplementary).

Whenever gene mutation frequencies per histological type or subtypes were not provided, their calculation was performed based on the formula:

$$\text{Mutated}\;\text{frequency}\;\text{of}\;\text{gene}\;X=\frac{\text{Nº}\;\text{of}\;\text{cases}\;\text{of}\;a\;\text{specific}\;\text{histological}\;\text{subtype}\;\text{mutated}\;\text{for}\;\text{gene}\;X}{\text{Total}\;\text{Nº}\;\text{of}\;\text{cases}\;\text{of}\;a\;\text{specific}\;\text{histological}\;\text{subtype}}$$

**Fig. 3:**
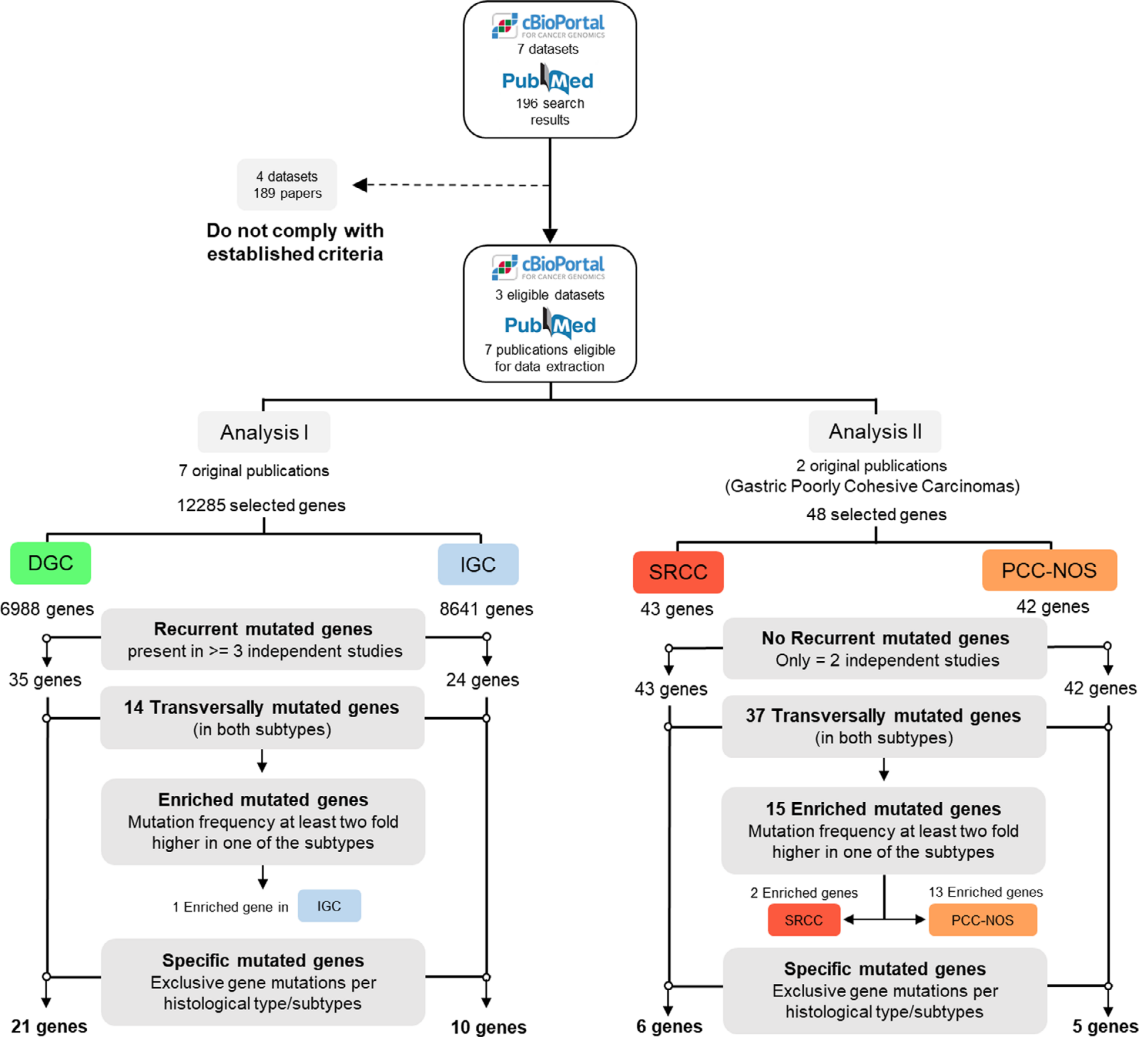
Data collection and research strategy for recurrent gene analysis.

According to criteria 1, seven different entries were identified; from these, only three data sets were used due to the availability of Laurén histological classification and specific gene mutation frequencies. According to criteria 2a, there were 162 results from which five publications were selected after manual curation. Criteria 2b retrieved 11 results, from which two had already been found with previous criteria and eight did not comply with our criteria after manual curation; therefore, only one publication was selected. Criteria 2c retrieved 29 results, from which four had already been found with previous criteria and 24 did not comply with our criteria after manual curation. Accordingly, a single new publication was selected based on criteria 2c. Overall, 3 data sets and 7 original articles were selected (Fig. 3).

All remaining articles not fulfilling the above criteria were excluded.

### 2.2 Criteria for recurrent gene analysis

To identify relevant mutated genes, we established two selection steps: gene recurrence and gene classification.

#### 2.2.1 Gene Recurrence

Only genes found to be mutated in at least three studies/resources in a frequency higher than 5% were selected for further analysis (Fig. 3, Analysis I). Due to the limited number of studies available for the analysis of SRCC and PCC‐NOS, all genes with an available mutation frequency were used for this part of the study (Fig. 3, Analysis II).

#### 2.2.2 Gene Classification

Mutated genes, for each histological type or subtype, were allocated to three classes: specific mutated genes, transversally mutated genes and enriched mutated genes.

Specific mutated genes were those exclusive of a single histological type or subtype.

Transversally mutated genes were those for which the difference between histological types or subtypes was, on average, lower than twofold. If the mutation frequency of a gene was reported in one study only, hampering average calculation, the absolute mutation frequency was used.

Enriched mutated genes were those for which difference between histological types or subtypes was, on average, higher than twofold. If the mutation frequency of a gene was reported in one study only, hampering average calculation, the absolute mutation frequency was used.

### 2.3 Identification of biological processes and molecular function associated with recurrent gene analysis

Specific mutated genes, transversally mutated genes and enriched mutated genes were used to feed Jvenn [58], an interactive diagram viewer, and allowed identifying genes overlapping between distinct GC histological types and subtypes through Venn Diagrams.

Recurrently mutated genes in DGC and IGC were compared to define the specific mutated genes for each histological type. The remaining DGC‐associated genes were considered transversally DGC‐ and IGC‐mutated genes, as no enrichment was seen in any of them. For the SRCC vs PCC‐NOS analysis, specific mutated genes per subtype were obtained from a single report.

Further, the output of the Venn diagrams was used to analyse Gene Ontology (GO) terms, biological processes and molecular functions associated with each gene set using Enrichr [59].

### 2.4 Search for clinical trials directed to DGC

Relevant clinical trials directed specifically to DGC patients were selected by searching the website www.clinicaltrials.gov (/clinicaltrials.gov/), and using as query terms ‘diffuse gastric cancer’, which retrieved 24 trials; ‘signet‐ring cell carcinoma’ with 19 trials; and ‘gastric poorly cohesive carcinoma’ with no results. After identifying only those associated with GC, 12 studies were selected. Only two clinical trials (NCT01285557 – DIGEST; NCT03977220 – NORDICA) were specifically directed to DGC patients, and one was directed to patients bearing SRCC (NCT01717924 – ADCI002 Study). Beyond these, and given the promising results recently obtained using anti‐claudin 18.2 antibodies, we manually added the MONO study where almost 50% of patients enrolled had DGC (NCT01197885 – MONO) [60]. In total, 13 clinical trials were selected since they were directed to DGC patients.

For these, a deeper analysis on therapeutic strategy, candidate drugs and molecular targets was performed.

### 2.5 Study limitations

There are several limitations in this study, namely the discrepancies between mutational profiles and frequencies among cohorts, which may be due to several factors: the methodological approach applied by different research groups (sequencing methods and pipeline analyses); the heterogeneity of GC (intra‐ and inter‐tumoral); and the sample size of the cohorts under analysis (specifically in the studies focused on SRCC and PCC‐NOS). Another issue is the lack of diversity in the geographical origin of the cohorts used in the selected studies for analysis, which reflects data available in the literature. However, the reproducibility of findings across the various molecular classifications (e.g. TCGA and ACRG) indicates that relevant GC‐associated molecular defects likely override population differences. Our analysis was restricted to mutational data, leaving behind transcriptomics and epigenetics, which can provide valuable data. Copy number variation analysis would have been a strength in this study; however, there are very few publications listing CNVs per case or their relative frequency to allow an accurate analysis. In addition, recent data suggested that copy number gains tended to occur more commonly in IGC than in DGC that was the focus of this work [61]. In addition, the comparison of sporadic and hereditary DGC suffered from scarce data published so far on somatic profiles in HDGC. Notwithstanding, we believe that the information herein provided constitutes a good basis for further studies aiming at a better understanding of the molecular pathogenesis of DGC and its clinical implications regarding prognosis and therapy.

## 3 Results

Strategies for gene mutation detection evolved from single‐gene analysis to the analysis of multigene panels, whole exomes or even whole genomes. This created the opportunity to derive multigene profiles that are valuable to correlate molecular defects with specific histological subtypes.

### 3.1 DGC vs IGC: distinctive mutational profiles

**Fig. 4:**
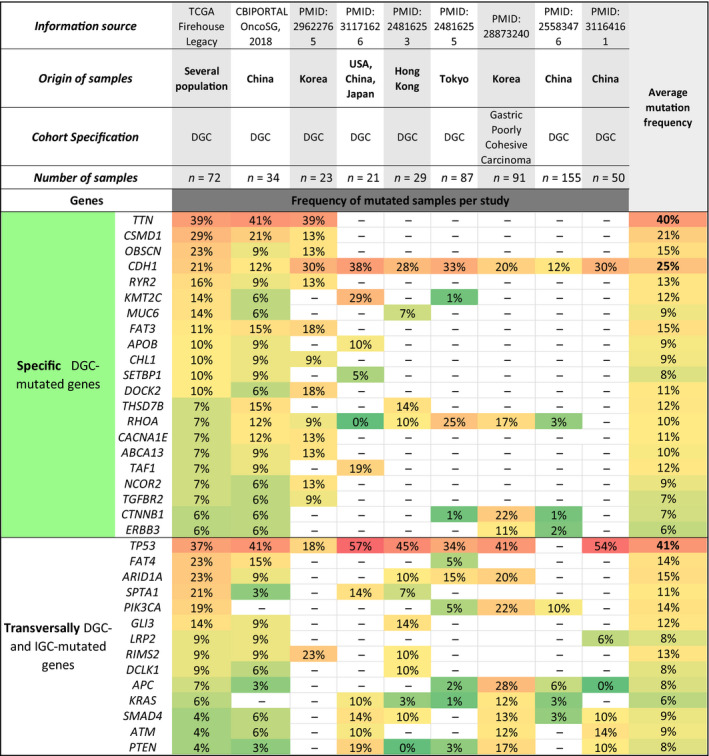
Mutational landscape of DGC subtype. Nine sources were used: two CBIOPORTAL data sets and seven references. The origin, number of samples, reported study identification and other cohorts’ characteristics are indicated in the heading of the table. The mutated genes of each subtype were divided into two main classifications: transversally mutated and specific genes. The specific mutated genes for DGC were associated with the green colour and are listed in the upper part of the table. In the lower part of the table, the transversally mutated genes in both DGC and IGC are presented. Mutation frequency was represented in a colour scale. Green was appointed to genes with mutation frequencies between 0% and 9% (less frequently mutated). Yellow was used for mutation frequencies between 10% and 19%. Light orange to mutation frequencies between 20% and 39%. A variation between dark orange and red was associated with mutation frequencies increasing from 40% to a maximum of 57% (frequently mutated). The average mutation frequency calculated for each DGC recurrent gene is present in the right column, and a similar colour scale was applied to distinguish the genes with higher mutation frequencies.

**Fig. 5:**
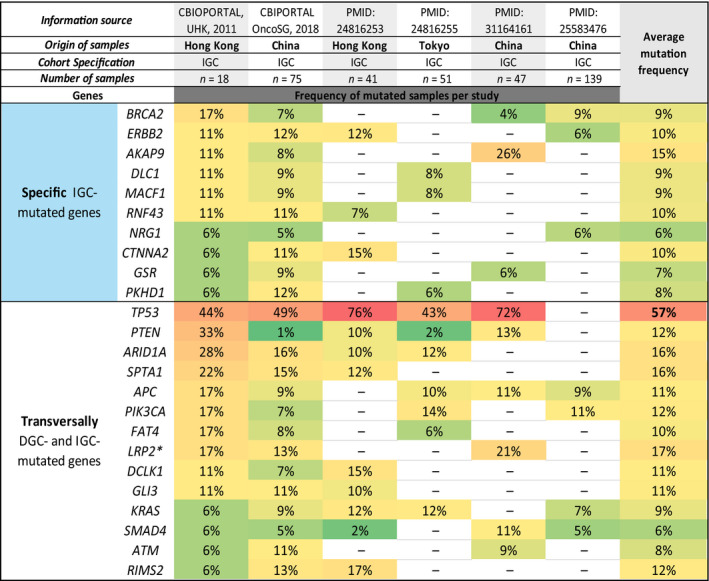
Mutational landscape of IGC subtype. Six sources were used: two CBIOPORTAL data sets and four publications. The origin, number of samples, reported study identification and other cohorts’ characteristics are indicated in the heading of the table. The mutated genes of each subtype were divided into two main classifications: transversally mutated and specific genes. The specific mutated genes for IGC were associated with the blue colour and are listed in the upper part of the Table. In the lower part of the table are presented the transversally mutated genes in both DGC and IGC. Mutation frequency was represented in a colour scale. Green was appointed to genes with mutation frequencies between 0% and 9% (less frequently mutated). Yellow was used for mutation frequencies between 10% and 19% and light orange for mutation frequencies between 20% and 39%. A variation between dark orange and red was associated with mutation frequencies increasing from 40% to a maximum of 76% (frequently mutated). The average mutation frequency calculated for each IGC recurrent gene is present in the right column, and a similar colour scale was applied to distinguish the genes with higher mutation frequencies. * LPR2 is enriched in IGC.

The data compilation performed herein and depicted in Fig. 4 shows the mutational landscape across DGC cohorts retrieved from nine independent studies, mainly Asian, and obtained with different technical approaches (four targeted sequencing; four WES, two WGS) (Data S1, Table S1). We next retrieved data from six independent studies of IGC, mostly also of Asian origin, having a similar degree of detailed information, depicted in Fig. 5. The mutational landscape of IGC obtained with different technological approaches (three targeted sequencing; three WES, two WGS) (Data S1, Table S1).

To derive the mutational landscape from DGC, we performed a recurrent gene analysis, as described in the methodology section that provided three mutated gene sets, both for DGC and for IGC: specific mutated genes, transversally mutated genes and enriched mutated genes.

### 3.2 DGC‐specific mutated genes

This analysis confirmed *CDH1* genetic variants as the hallmark of the DGC histological type [31, 33, 34], as *CDH1* is the only gene found to be mutated in all DGC data sets and in none of the IGC data sets (specific DGC‐mutated gene) (Figs 4 and 5). *CDH1* variants were found on average in 25% of DGC cases, ranging from 12% to 38% among studies (Fig. 4). Besides *CDH1*, another 20 genes were found to be mutated specifically in DGC, in at least three studies (Fig. 4). *TTN* gene mutations were found on average in 40% of DGC cases, and described in three studies, making this gene the top‐ranking specific DGC‐mutated gene, while *RHOA* and *CTNNB1* were found to be mutated in seven and five studies, on average in 10% and 7% of the cases, respectively. All other specific DGC‐mutated genes were found in three or four studies (Fig. 4). Seven of these were mutated on average in > 12% of DGC. *FAT3* (15%) and *KMT2C* (12%) have a previously reported association with DGC; *CSMD1* (21%), *OBSCN* (15%), *RYR2* (13%) and *TAF1* (12%) have been previously associated with GC but not DGC, and *THSD7B* (12%) with no previous association with GC.

There are other specific DGC‐mutated genes depicted in Fig. 4 that present on average < 12% of mutations that we used for downstream analyses.

### 3.3 IGC‐specific mutated genes

We next focused on the mutational landscape of IGC from six studies, to identify the set of specific IGC‐mutated genes (Fig. 5). No single gene was commonly mutated across all IGC data sets; however, *BRCA2* and *ERBB2* were specific IGC‐mutated genes (9% and 10% on average, respectively), according to four out of six studies.

Additionally, *AKAP9*, *DLC1*, *MACF1*, *RNF43*, *NRG1*, *CTNNA2*, *GSR* and *PKHD1* were also identified as specific IGC‐mutated genes. *AKAP9* (found in 3/6 studies) was the one with the highest average mutation frequency (15%).

### 3.4 Transversally DGC‐ and IGC‐ mutated genes

We then ranked the transversally DGC‐ and IGC‐mutated genes across 3 studies or more (Figs 4 and 5). These were *TP53*, *FAT4*, *ARID1A*, *SPTA1*, *PIK3CA*, *GLI3*, *LRP2*, *RIMS2*, *DCLK1*, *APC*, *KRAS*, *SMAD4*, *ATM* and *PTEN* (Figs 4 and 5). From these 14 genes, a single gene was classified as enriched mutated gene in IGC (*LRP2*), as its mutation frequency was >twofold higher in IGC (17%) as compared to DGC (8%) (Figs 4 and 5). All remaining genes displayed similar mutation frequencies (<twofold difference between histotypes) in both DGC and IGC.

*TP53* and *ARID1A* genes were the most frequently transversally mutated genes across DGC (41% and 15%, respectively) and IGC (57% and 16%, respectively) data sets (Figs 4 and 5). Besides these two important GC driver genes, *APC* and *PTEN* tumour suppressor driver genes, as well as *KRAS* and *PIK3CA* driver oncogenes [62], were also transversally mutated in both GC histotypes with frequencies below 15% across data sets (Figs 4 and 5).

### 3.5 DGC vs IGC: biological processes and molecular functions associated with mutational profiles

**Fig. 6:**
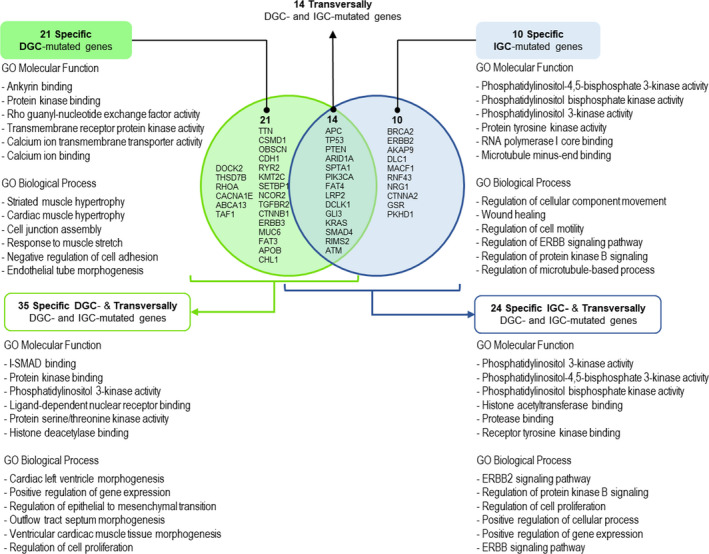
Integrative analysis of specific‐ and transversally mutated genes in DGC or IGC. GO terms for molecular functions and biological processes are depicted for the specific genes lists and for the overall set of genes of each subtype (specific‐ and transversally mutated genes analysed together). The analysis generated using Enrichr and the associated GO terms number is available in Data S2.

The comparison of molecular functions and biological terms derived from the mutational landscapes of specific DGC genes vs specific IGC genes (Fig. 6) shows that the recurrence and high mutation frequency of *CDH1* and *RHOA* associate DGC with cell adhesion and calcium dependence. On the other hand, specific IGC‐mutated genes are mainly associated with tyrosine kinase activity and related signalling pathways, encompassing some promising therapeutic targets (Table 2).

As tumour cells most likely bear histotype‐specific mutations together with histotype nonspecific mutations, equivalently frequent, we combined the specific DGC‐mutated gene set with the transversally DGC‐ and IGC‐mutated gene set (all genes listed in Fig. 4) and compared the resulting gene list with its IGC counterpart (all genes in Fig. 5) regarding molecular functions and biological terms. This integrated analysis of specific and transversally mutated genes, which is depicted in Fig. 6, turned DGC closer to IGC, with proliferation and tyrosine kinase activity and associated signalling pathways appearing as an output, also in DGC. However, regulation of epithelial‐to‐mesenchymal transition was still a feature of DGC only. This analysis also revealed the involvement of histone modifications associated with both histological types, histone deacetylase related to DGC and histone acetyltransferase (HAT) to IGC. Details on all analyses are presented in Fig. 6 and Data S2.

### 3.6 SRCC vs PCC‐NOS: distinctive mutational profiles

We then wanted to understand whether the mutational landscape of DGC is common across DGC histological subtypes, or if each subtype (SRCC and PCC‐NOS) has its unique molecular profile.

For this, we compared the set of genes mutated in SRCC and in PCC‐NOS, described in two Asian studies and obtained with different technical approaches (one targeted sequencing; one WES) (Data S1, Table S1).

### 3.7 SRCC vs PCC‐NOS transversally mutated genes

**Fig. 7:**
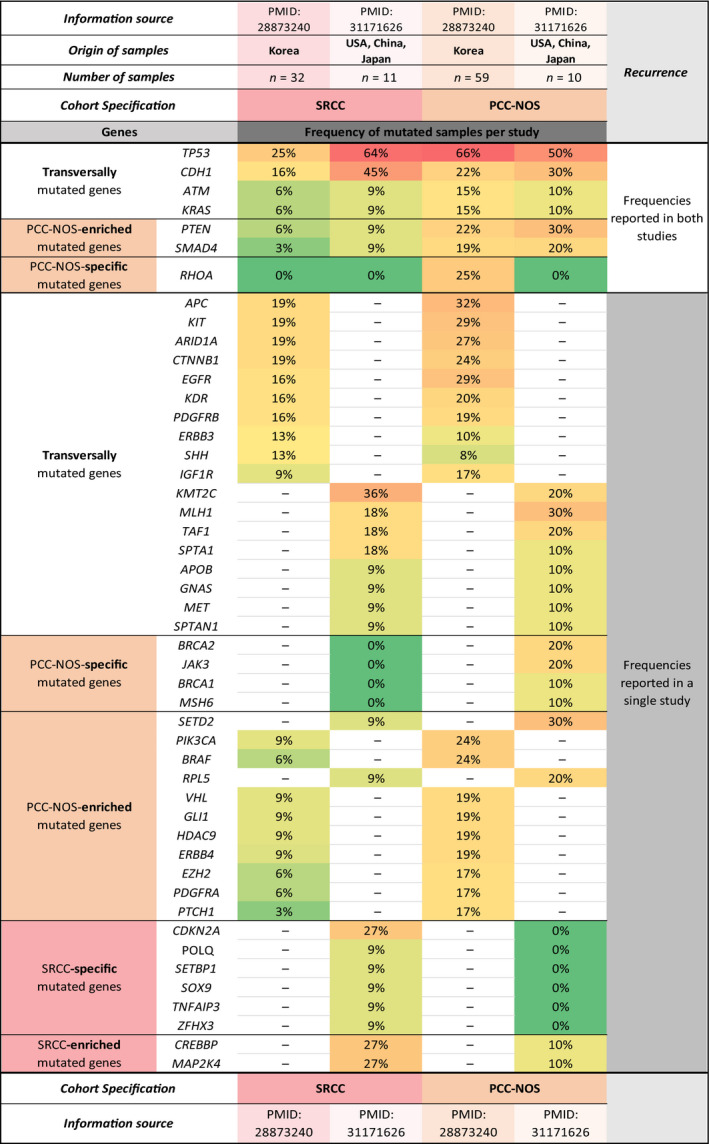
Mutational landscape of SRCC and PCC‐NOS. For this analysis, two original publications were used. The origin, number of samples, reported study identification and other cohorts’ characteristics are indicated in the heading of the table. The mutated genes of each subtype were divided into three main classifications: enriched, transversally mutated and specific genes. Each mutation frequency was associated with a colour scale. Green was appointed to genes with mutation frequencies between 0% and 9% (less frequently mutated). Yellow was used for mutation frequencies between 10% and 19% and light orange for mutation frequencies between 20% and 39%. A variation between dark orange and red was associated with mutation frequencies increasing from 40% to a maximum of 66% (frequently mutated).

There were 22 transversally mutated genes across SRCC and PCC‐NOS, from which four genes (*CDH1*, *TP53*, *ATM* and *KRAS)* were found in two independent data sets per histological subtype (Fig. 7). *TP53* was the most frequently mutated gene in both DGC subtypes. The remaining transversally mutated genes in SRCC and PCC‐NOS were found in a single study, and none of them was mutated >twofold when comparing SRCC and PCC‐NOS (Fig. 7).

### 3.8 SRCC‐ vs PCC‐NOS‐enriched mutated genes

Only two genes (*CREBBP* and *MAP2K4*) were found enriched in SRCC (27% for both) as compared to PCC‐NOS (10% for both) (Fig. 7). On the other hand, 13 mutated genes were found enriched in PCC‐NOS as compared to SRCC. From these, *PTEN, SMAD4*, *SETD2*, *PIK3CA*, *BRAF* and *RPL5* mutations were found in at least 20% of PCC‐NOS as compared to a maximum of 9% in SRCC (Fig. 7). Most PCC‐NOS enriched mutated genes either had tumour suppressor activity or were associated with histone markers of transcription repression.

### 3.9 SRCC‐specific mutated genes

Six genes (*CDKN2A*, *POLQ*, *SETBP1*, *SOX9*, *TNFAIP3* and *ZFHX3*) were found to be exclusive of SRCC (SRCC‐specific mutated genes) (Fig. 7). While *CDKN2A* was found mutated in 27% of the cases, the remaining five genes were found mutated in < 10% of the SRCC.

### 3.10 PCC‐NOS‐specific mutated genes

Five genes (*RHOA*, *BRCA2*, *JAK3*, *BRCA1* and *MSH6*), were found exclusively mutated in PCC‐NOS (PCC‐NOS‐specific mutated genes) (Fig. 7). While *RHOA*, *BRCA2* and *JAK3* were found mutated in > 20% of the cases, *BRCA1* and *MSH6* genes were found mutated in 10% of PCC‐NOS cases.

This mutation frequency‐focused analysis revealed more similarities than differences between SRCC and PCC‐NOS, regarding the set of genes affected in both subtypes. However, some very specific features were also found per subtype, likely supporting their morphological differences.

### 3.11 Insights into similarities and differences between SRCC and PCC‐NOS mutational profiles

**Fig. 8:**
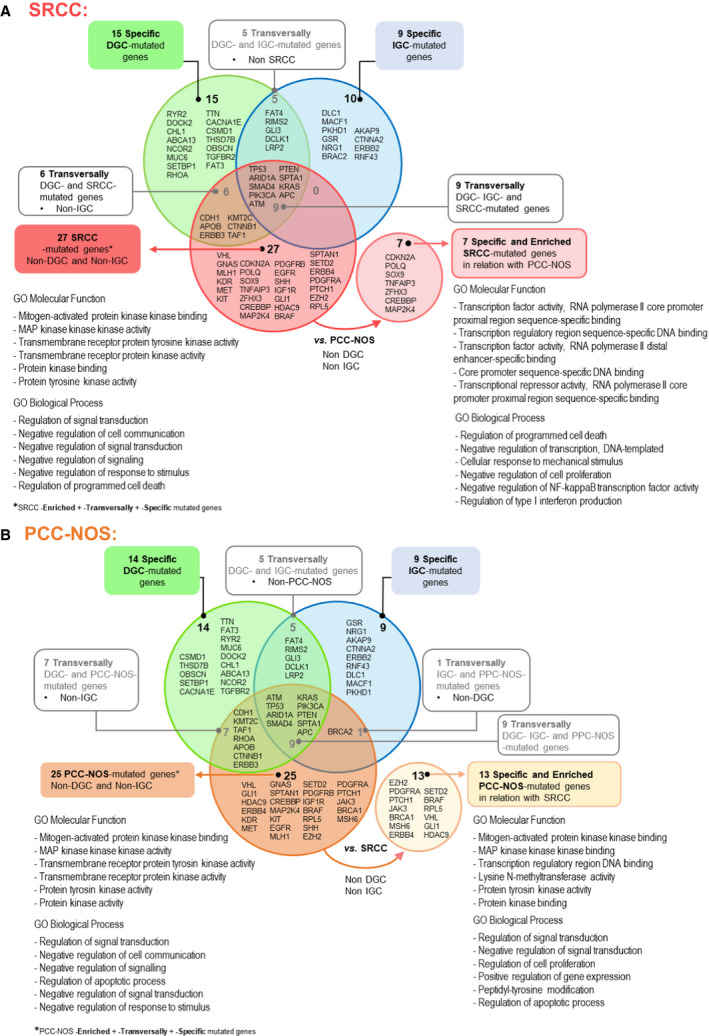
Specific and transversally mutated genes in SRCC (A) and PCC/NOS (B) in comparison with DGC and IGC. SRCC‐associated genes were linked to a red colour, while PCC‐NOS‐associated genes are related to the orange colour. In both Fig. 8 A, B, the circle outside of the Venn diagram contains the specific genes for SRCC and PCC‐NOS, respectively. GO terms for molecular functions and biological processes are depicted for the mutated genes associated with each subtype that were not shared with DGC nor IGC, as well for the SRCC/PCC‐NOS‐specific genes. The analysis generated using *Enrichr* and the associated GO terms number is available in Data S2.

We compared the mutation landscapes of DGC, IGC, SRCC and PCC‐NOS aiming to address particular mutational landscapes of SRCC and PCC‐NOS. Nine genes were shared by DGC, IGC, PCC‐NOS and SRCC. These are well‐known cancer driver genes with crucial functions related to cell proliferation, migration, DNA repair or angiogenesis [63]. Some are well‐known tumour suppressors such as *TP53*, *ATM*, *APC*, *ARID1A, SMAD4* and *PTEN* or oncogenes such as *PIK3CA* and *KRAS* (Fig. 8A,B).

SRCC shares a set of six mutated genes with PCC‐NOS, which are also found in DGC but not in IGC (*CDH1, APOB, ERBB3, KMT2C, CTNNB1, TAF1*) (Fig. 8A,B). Among these six genes is the hallmark gene *CDH1* (Fig. 8A,B). There are no genes shared between SRCC and IGC, and *BRCA2* is the only mutated gene shared by PCC‐NOS and IGC (Fig. 8B). These data likely identify the mutated genes that are common in DGC, SRCC and PCC‐NOS, in keeping with the fact that the two latter are subtypes of the former. SRCC and PCC‐NOS diverge in morphology and also in their mutational profile. We found seven genes that are SRCC‐specific and enriched genes (*CDKN2A, POLQ, SOX9, TNFAIP3, ZFHX3, CREBBP and* *MAP2K4*) in relation to PCC‐NOS (Fig. 8A). Both in the restricted set of seven specific and enriched SRCC‐mutated genes and in a larger set of 27 SRCC‐mutated genes (Fig. 8A), an association with programmed cell death is always identified among GO biological processes, representing a novel feature of SRCC. Moreover, the seven genes that differentiate SRCC from all other GC histotypes or subtypes are mainly associated with transcriptional activity (TOP6 ranking GO molecular functions) and cell proliferation and immune or inflammatory response (TOP6 GO biological processes) (Fig. 8A).

We found 25 mutated genes that differentiate PCC‐NOS from DGC and IGC, and from these, 13 are PCC‐NOS‐specific and enriched genes (*EZH2, SETD2, PDGFRA, BRAF, PTCH1, RPL5, JAK3, VHL, BRCA1, GLI1, MSH6, HDAC9 and ERBB4*) in relation to SRCC (Fig. 8B). This combination of mutated genes likely represents a distinctive feature of PCC‐NOS, which despite being mainly associated with MAPK pathway and protein kinase activity, as for the main GC histotypes, it is specifically associated with transcription regulatory region DNA binding (TOP6 ranking GO molecular functions). This feature brings PCC‐NOS closer to SRCC (Fig. 8B).

In summary, this analysis supports a divergent evolution of SRCC and PCC‐NOS mutation profiles and highlights particular molecular pathways and functions from each of these two DGC subtypes.

### 3.12 Therapies specifically directed to DGC do not consider DGC mutational landscape

We searched for ongoing and recently closed clinical trials specifically mentioning the inclusion of patients with DGC or DGC subtypes to understand whether any of these trials used as therapy target, the genes or pathways herein identified has preferentially involved in DGC and its subtypes.

We found 13 clinical trials using the search criteria described in the methodology (Table S2). In total, we found four trials directed to DGC patients, two completed (DIGEST and MONO) and two currently recruiting or about to start recruitment (NORDICA and ADCI002 Study) (Table 3).

**Table 3:** Details of clinical trials registered at ClinicalTrials.gov directed to DGC patients.Green was used to highlight clinical trials that specifically mention DGC; red was used to highlight clinical trials that specifically mention SRCC; and white was used to highlight a specific clinical trial with promising results based on CLDN18.2 expression (Table 2).

ClinicalTrials.gov Identifier	Title of the clinical trial	Strategy	Molecular target	Status	Publications	Clinical trial outcome
NCT03977220	Nab‐paclitaxel combined with S‐1 treating diffuse type of stage Ⅲ gastric cancer as adjuvant setting (NORDICA)	Evaluate paclitaxel for microtubules stabilization and S‐1 (oral derivative of 5‐FU)	Microtubules and conventional chemotherapy targets	Not yet recruiting	No publications available	Not available
NCT01717924	Evaluation of Surgery vs Primary Chemotherapy in Resectable Signet‐ring Cell Gastric Adenocarcinoma (ADCI002) (Phase II/III)	Compare primary surgery vs primary chemotherapy followed by surgery. epirubicin for topoisomerase II inhibition; cisplatin; 5‐fluorouracil	Topoisomerase II and conventional chemotherapy targets	Recruiting	[65]	Not available
NCT01285557	Diffuse Gastric and Esophagogastric Junction Cancer S‐1 Trial (DIGEST) (Phase III)	Evaluate the safety and efficacy of S‐1 and cisplatin compared with 5‐FU and cisplatin	Conventional chemotherapy targets	Completed	[64]	No significant difference has been found in the outcome of the patients comparing both approaches
NCT01197885	Efficacy and Safety Study of Multiple Doses of IMAB362 in Patients with Advanced Gastroesophageal Cancer (MONO) (Phase Iia)	Evaluate the safety and efficacy of IMAB362 used as monoclonal antibody against CLDN18.2 (single agent) in GC patients (22/54 DGC)	CLDN18.2	Completed	[60]	Zolbetuximab was well‐tolerated and exhibited antitumour activity with a clinical benefit rate for patients of 23%; whether these were DGC patients was not disclosed

The DIGEST phase III study (NCT01285557) compared the use of fluorouracil (5‐FU) combined with cisplatin with the use of the latter plus S‐1 in 361 patients with metastatic DGC (Table 3). This trial has been completed, and results showed that there was no significant difference in the outcome of the patients comparing both approaches [64].

The MONO study (NCT01197885), a phase II clinical trial, examined the outcome of zolbetuximab (monoclonal antibody against CLDN18.2: IMAB362) monotherapy in a series of patients with recurrent or refractory, locally advanced or metastatic and CLDN18.2‐positive gastric, gastro‐oesophageal junction, or oesophageal adenocarcinoma (Table 3). From the 54 patients enrolled in this trial, 22 were advanced DGC patients with CLDN18.2 expression in ≥ 50% of tumour cells. Zolbetuximab was well‐tolerated and exhibited antitumour activity with a clinical benefit rate of 23% [60]; whether patients benefiting from this targeted therapy were DGC patients is unknown.

The ADCI002 phase II/III clinical trial (NCT01717924), that is currently recruiting, was designed to address the inherent chemoresistance of SRCC. It evaluates primary surgery vs primary chemotherapy followed by surgery, using epirubicin for topoisomerase II inhibition combined with cisplatin and 5‐FU [65].

The NORDICA phase I study (NCT03977220), which is specifically directed to DGC patients, is not yet recruiting and aims at using microtubules stabilization agent (paclitaxel) combined with S‐1 (Table 3).

None of the trials described above used as target for therapy, the specific mutations or mutation profiles and associated molecular functions herein found to differentiate DGC and its subtypes from each other and from IGC.

### 3.13 Sporadic DGC and hereditary DGC (HDGC): somatic mutational profiles

We tried to understand whether DGC arising in different settings (hereditary or sporadic) would share a similar mutational profile, and whether DGC arising in the HDGC context would mimic a mutational profile closer to that of SRCC.

The so‐called HDGC syndrome is caused mainly by germline *CDH1* and rarely by *CTNNA1* pathogenic or likely pathogenic variants and is dominated by the development of early‐onset DGC, mainly SRCC [66, 67, 68]. However, the somatic mutational landscape of DGC/SRCC arising in this setting is, to date, fairly unknown.

We could only find two studies reporting the somatic mutational landscape of HDGC tumours [67, 69]. The germline causative defect in one family affected *CDH1* [69] and in the other family affected *CTNNA1* [67].

We compared the list of somatic mutated genes found in these HDGC cases with that found in sporadic DGC cases (Fig. 4). The mutational profile of the *CTNNA1* germline mutated tumour [67] encompassed somatic mutations in *RHOA*, a specific DGC and PCC‐NOS gene (Fig. 7), and in *ARID1A* and *PIK3CA*, genes transversally mutated in both DGC and IGC (Fig. 7). This suggests that, independently of the cancer setting (hereditary vs diffuse), or histological type (DGC vs IGC), GC developing in this *CTNNA1* variant germline carrier presents a nonspecific combination of somatic events. By contrast, for the *CDH1* germline mutated tumour, somatic mutations were described in *TTN* and *CDH1* by Funakoshi *et al* [69], which are both specific DGC‐mutated genes (this study, Fig. 6); being *CDH1* was also considered transversally mutated in SRCC and PCC‐NOS (Fig. 7).

To better understand whether there are real differences between the mutational profiles of sporadic and hereditary DGC, or whether HDGC cases are closer to a particular DGC subtype, additional studies need to be performed.

## 4 Discussion

In the present study, we started by revising GC histological and molecular classifications, highlighting associated relevant molecular landscapes, clinical outcomes and currently approved or promising therapies. The state of the art on all these aspects is presented in the introduction section. The main outcome of this literature revision was the identification of an asymmetry, regarding prognosis, therapy targets and available effective therapies (both conventional and targeted), in TCGA GS and ACRG MSS/EMT, mainly encompassing DGC, in comparison with other molecular subgroups.

To address this gap in knowledge, and shed light into potential targets for therapeutic intervention, we explored a set of studies reporting on multigene mutational analysis to identify DGC‐associated mutational profiles, molecular pathways and biological functions. We chose this approach, as mutational profiles are rather stable genetic events, easy to detect, interpret and less costly as compared to RNA profiles, methylation patterns or other epigenomic approaches. On the other hand, several tumour‐specific mutations constitute well‐proven, highly sensitive and specific predictive biomarkers of response to selective targeted therapies and are now an indispensable laboratory tool for therapeutic decisions [70].

Our systematic analysis confirms that *TP53, ARID1A, SMAD4, PIK3CA, PTEN, KRAS, APC, ATM and SPTA1* are driver and central genes in gastric tumorigenesis, and transversal to the main GC histotypes and DGC subtypes [62].

Also, herein we identified specific sets of genes that define the main molecular pathways classically involved in either DGC or IGC. However, tumours emerge and evolve due to a mutational profile that can be shared with other tumour types. We could find that the combination of all recurrently mutated genes either in DGC or in IGC triggered the involvement of similar molecular pathways regardless of being histotype‐specific or common to both histotypes. This may explain why conventional therapies, and even some approved targeted therapies, demonstrate clinical benefit regardless of the GC histological type.

We further showed that the most recurrently mutated and specific genes in DGC, across many studies, were *CDH1* and *RHOA*. This result is reflected in the findings from the TCGA GS and ACRG MSS/EMT subgroups, which are dominated by DGC. According to both studies, this subgroup presents the worst prognosis within GC molecular subtypes, resistance to conventional chemotherapy and so far, have no approved targeted therapies [22, 37, 49]. Interestingly, a recent study reported that late‐onset DGCs presented higher mutation frequencies in *RHOA* and less frequent mutations in *CDH1* in comparison with early‐onset DGCs [31, 71].

Although none of the ongoing clinical trials directed to DGC patients use these two proteins as targets for therapy (Table 3), it is tempting to speculate whether their mutations would perform well as therapy targets or predictors of therapy responsiveness. E‐cadherin inactivation is a highly unlikely druggable target mechanism, and it has been shown to cause multidrug resistance. By contrast, E‐cadherin disruption is also known to induce upregulation of the anti‐apoptotic protein BCL2, and activation of Rho‐Rock pathway, which may be targetable (reviewed in Ref. [72]). Taking advantage of the pro‐survival context in E‐cadherin deficient DGC, treatment using pro‐apoptotic drugs may be feasible. Indeed, preclinical treatment with pro‐apoptotic BH3‐only mimetics combined with chemotherapy or endocrine treatment regimens had a positive effect in breast cancer [73, 74]. Preclinical studies also suggested that pharmacological inhibition of Rho‐Rock signalling may be a clinically relevant target in cancer [75]. Other genes specifically mutated in DGC may also be interesting to consider when rethinking biomarkers for prognosis and therapy in patients with DGC. An example is the *TTN* gene, whose mutations were found specifically in DGC and on average in 40% of the cases. Although not previously related specifically to DGC, *TTN* was found as one of the top five hub genes of a specific co‐expression module positively correlated with GC pathological tumour and lymph node stages [76]. In another study, *TTN* gene mutations were correlated with poor prognosis and predicted tumour mutation burden and immunotherapy efficacy in GC [77]. Other examples are *FAT3* and *KMT2C*, herein found mutated on average in 15% and 12% of DGC, respectively, and found previously associated with this cancer histotype [76, 78, 79]. Namely, *KMT2C* loss was shown to promote epithelial‐to‐mesenchymal transition and was associated with poor overall survival.

Although our study has not addressed the role of epigenetic changes in DGC, these events are also considered significant carcinogenic factors, and epigenetic regulation has been claimed as a promising molecular target for therapy in GC precision oncology [17, 80]. Herein, we have shown that histone modifications appear not only in the TOP6 molecular functions from DGC, but also in those from IGC, and when specific and transversally mutated genes are considered. This finding reinforces the need of studies focusing specifically on the epigenetics profiles of DGC and its subtypes, with the aim of finding potential targets for precision oncology.

The current systematic analysis also provides, to our knowledge the first combined analysis on SRCC and PCC‐NOS mutational profiles, considering two independent studies. This revealed *CDH1*, *TP53*, *ATM* and *KRAS* mutations as recurrent and common events of both DGC subtypes, again supporting the role of GC driver genes, and *RHOA* mutations as a specificity of PCC‐NOS [16]. In terms of more specific mutational landscapes, both sets of divergent mutated genes between overall DGC and SRCC and between overall DGC and PCC‐NOS are associated with TRK pathways, cell–cell communication and apoptosis, although involving different genes. However, and interestingly, the set of genes that distinguish SRCC and PCC‐NOS from DGC overall and from each other associates with transcriptional regulation and gene expression control, besides TRK pathways and apoptosis, particularly the SRCC subtype. Indeed, *CREBBP* and *MAP2K4* genes, whose mutations are enriched in SRCC as compared to PCC‐NOS (Fig. 7), are involved in coupling chromatin remodelling to transcription factor recognition and transcription regulation, respectively [81, 82]. A loss‐of‐function mutation in the *CBP/CREBBP* gene, which encodes a HAT, is common to many cancers, including GC [82], and *MAP2K4* loss of expression has been reported in gastric adenocarcinoma associated with poor survival [81]. Given that a great deal of transcriptional control involves epigenetic regulation, epigenetic‐related treatments may hold promise for SRCC and PCC‐NOS precision oncology [80], besides the above‐mentioned possibilities described for DGC overall.

To the best of our knowledge, this is the first systematic analysis describing the distinctive mutational profiles of DGC and its subtypes, resourcing to a recurrent analysis, to address the gap in potential treatment options for this GC histotype. By exploring the literature regarding clinical trials directed to the particularities of DGC, we concluded that these are only a few and rarely specifically designed for DGC patients. This may explain the delay in finding treatment options for these patients. Because we were not able to find more promising results than those obtained for anti‐Claudin18.2 targeted therapy, we herein highlight a set of recurrent mutant genes and associated pathways that may be explored for novel therapeutic designs in DGC and its subtypes. The interpretation of past clinical trials in the light of tumour morphology can also guide therapeutic development by identifying subsets of patients with better response to a certain treatment strategy. Exploring the mutational landscape of DGC subtypes can further identify new and more adequate druggable targets with therapeutic implications.

Finally, improving the knowledge on somatic molecular/genomic players in sporadic DGC may also be valuable to guide future treatments in the HDGC setting, which also remains poorly studied from the somatic standpoint and without chemotherapeutic options.

## Conflict of interest

The authors declare no conflict of interest.

## Author contributions

CO and FC conceived and supervised the study; CO, RB‐M and JG‐P designed the study; RB‐M, JG‐P and IG acquired the data; RB‐M, JG‐P, IG, FC and CO analysed and interpreted the data; all authors drafted the manuscript; FC, IG and CO edited and reviewed the manuscript; CO and FC obtained funding. All authors critically revised the manuscript for important intellectual content. All authors have read and agreed to the published version of the manuscript.

## Supporting information


**Data S1**. Full data on gene mutation frequencies obtained from the selected studies (sheet 1), reporting all mutated genes found for DGC cases (sheet 2), IGC cases (sheet 3) and SRCC/PCC‐NOS (sheet 4).

**Data S2**. GO TERMS, Biological Processes and Molecular Functions retrieved by Enrichr for each genes list: Transversally + Specific DGC/IGC, Specific DGC/IGC genes, Transversally + Enriched + Specific SRCC genes, Specific SRCC genes, Transversally + Enriched + Specific PCC‐NOS genes, and Specific PCC‐NOS genes.

**Table S1**. Sample size and details of sequencing methodologies used in the selected publications for analysis.

**Table S2**. Details of Clinical trial registered at ClinicalTrials.gov directed to gastric cancer and often including DGC patients.
