# Supplementary material for: Histological and mutational profile of diffuse gastric cancer: current knowledge and future challenges
Source: Mol Oncol. 2021 May 2;15(11):2841–67. doi: 10.1002/1878-0261.12948 (PMC8564639; doi:10.1002/1878-0261.12948)

File S2. GO TERMS, Biological Processes and Molecular Functions retrieved by Enrichr for each gene list.

Enrichr

Transversally + Specific DGC genes (35 genes)

DOCK2	MUC6	PIK3CA
THSD7B	FAT3	RIMS2
RHOA	APOB	GLI3
CACNA1E	CHL1	SPTA1
ABCA13	SETBP1	ATM
TAF1	NCOR2	SMAD4
TTN	TGFBR2	DCLK1
CSMD1	CTNNB1	PTEN
OBSCN	ERBB3	LRP2
CDH1	TP53	APC
RYR2	ARID1A	KRAS
KMT2C	FAT4	

Specific DGC genes (21 genes)

DOCK2	KMT2C
THSD7B	MUC6
RHOA	FAT3
CACNA1E	APOB
ABCA13	CHL1
TAF1	SETBP1
TTN	NCOR2
CSMD1	TGFBR2
OBSCN	CTNNB1
CDH1	ERBB3
RYR2	

Transversally + Specific IGC genes (24 genes)

BRCA2	FAT4
ERBB2	PIK3CA
AKAP9	RIMS2
DLC1	GLI3
MACF1	SPTA1
RNF43	ATM
NRG1	SMAD4
CTNNA2	DCLK1
GSR	PTEN
PKHD1	LRP2
TP53	APC
ARID1A	KRAS

Specific IGC genes (10 genes)

BRCA2
ERBB2
AKAP9
DLC1
MACF1
RNF43
NRG1
CTNNA2
GSR
PKHD1

Transversally + Enriched + Specific PCC-NOS genes non-DGC and non-IGC (25 genes)

VHL	PDGFRA
GLI1	PTCH1
HDAC9	JAK3
ERBB4	BRCA1
EZH2	MSH6
PDGFRB	MLH1
IGF1R	GNAS
KIT	MET
EGFR	SPTAN1
KDR	CREBBP
SHH	MAP2K4
SETD2	RPL5
BRAF	

Specific + Enriched PCC-NOS genes non-DGC and non-IGC (13 genes)

SETD2	JAK3
BRAF	BRCA1
RPL5	MSH6PO
VHL	LQ
GLI1	SOX9
HDAC9	TNFAIP3
ERBB4	ZFHX3
EZH2	CREBBP
PDGFRA	MAP2K4
PTCH1	

Transversally + Enriched + Specific SRCC genes non-DGC and non-IGC (27 genes)

POLQ	SPTAN1
SOX9	SETD2
TNFAIP3	ERBB4
ZFHX3	PDGFRA
CREBBP	PTCH1
MAP2K4	PDGFRB
KIT	EGFR
VHL	SHH
GNAS	IGF1R
MLH1	GLI1
KDR	HDAC9
MET	BRAF
EZH2	RPL5

Specific + Enriched SRCC genes non-DGC and non-IGC (7 genes)

CDKN2A
POLQ
SOX9
TNFAIP3
ZFHX3
CREBBP
MAP2K4

Specific SRCC genes (6 genes)

RHOA
BRCA2
JAK3
BRCA1
MSH6

Specific PCC-NOS genes (5 genes)

CDKN2A
POLQ
SETBP1
SOX9
TNFAIP3
ZFHX3

Specific DGC genes (21 genes)

GO Biological Process 2018

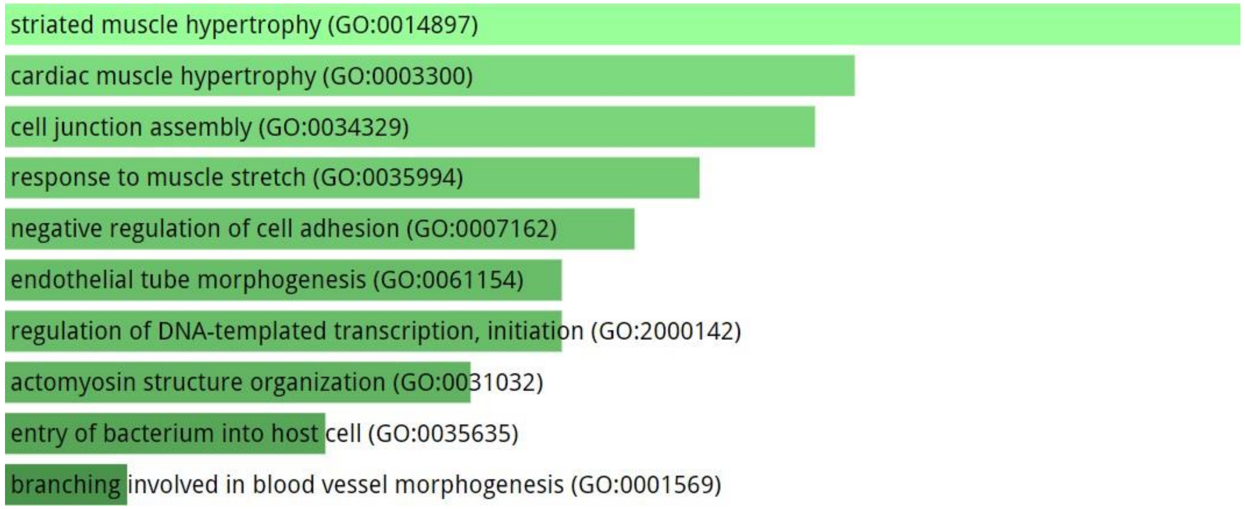

GO Molecular Function 2018

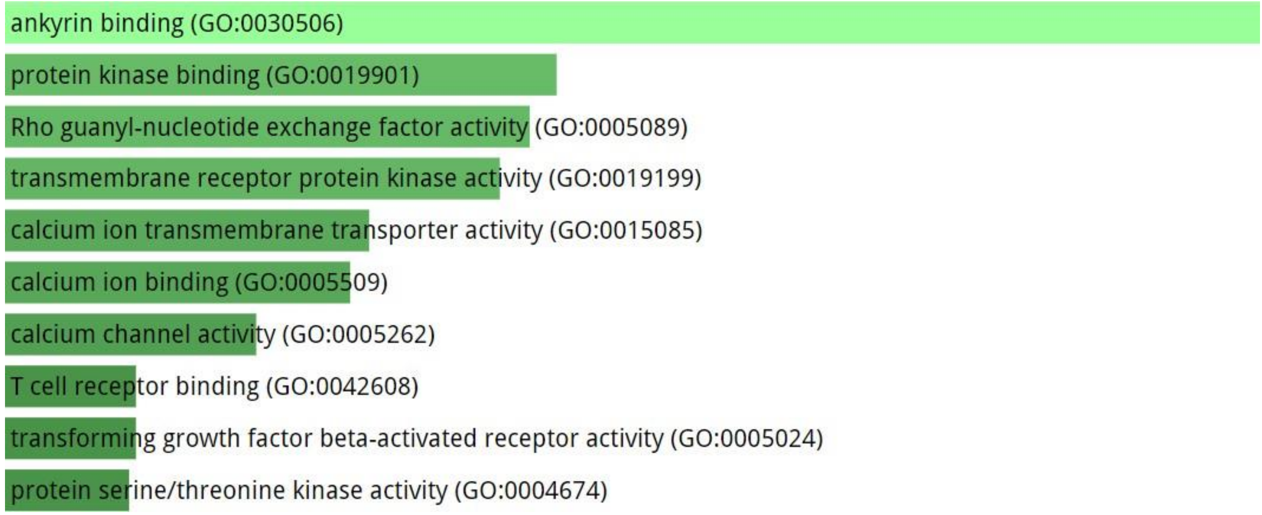

Specific IGC genes (10 genes)

GO Biological Process 2018

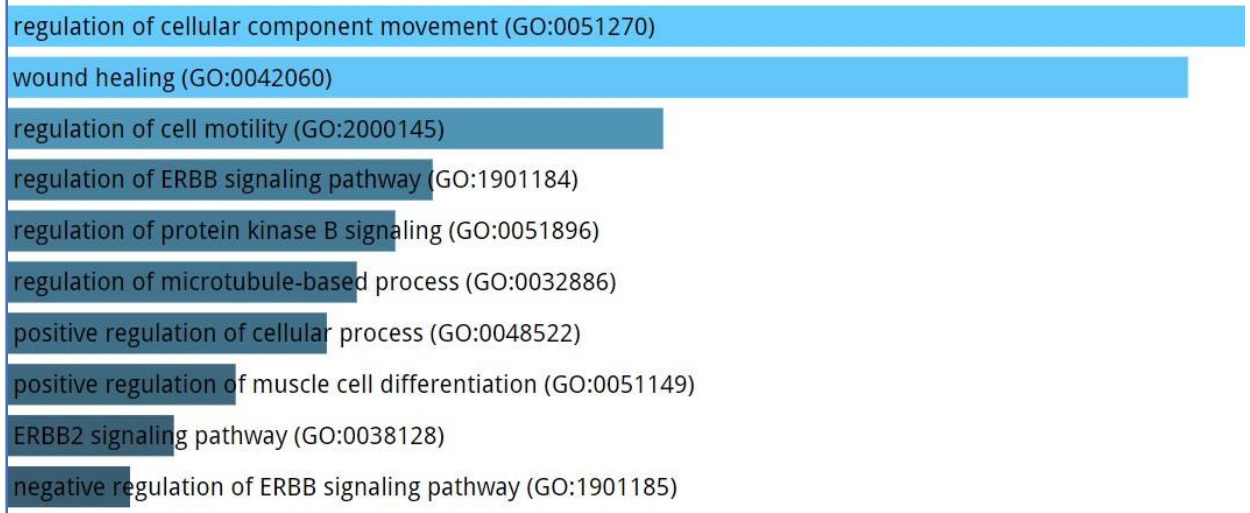

GO Molecular Function 2018

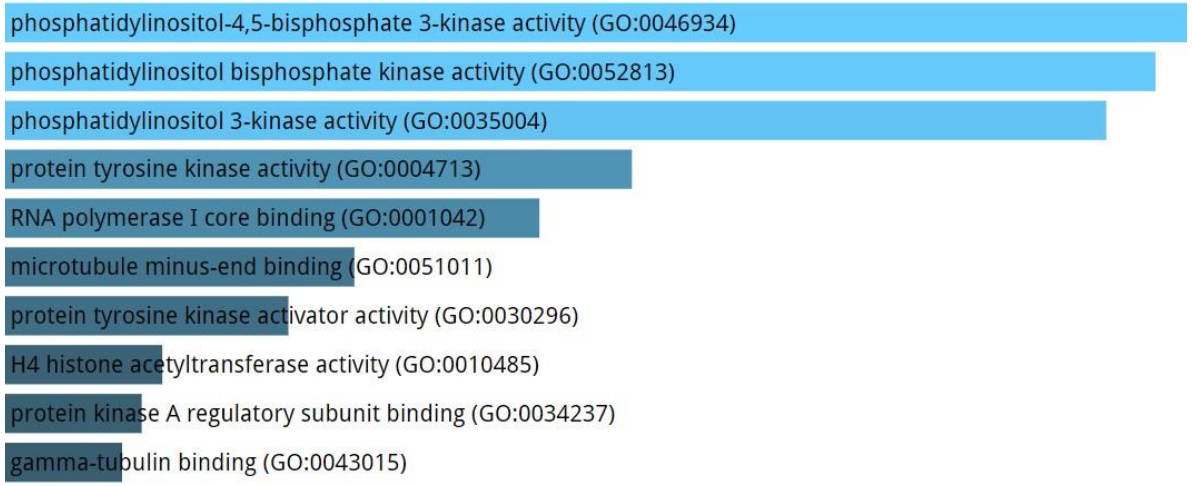

Specific + transversally DGC genes (35 genes)

GO Biological Process 2018

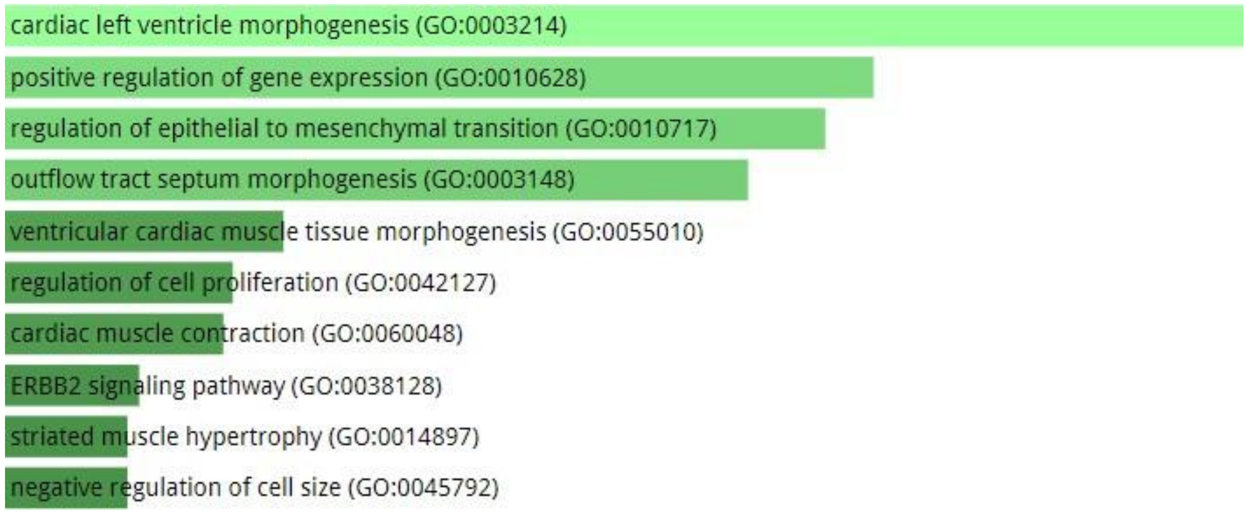

GO Molecular Function 2018

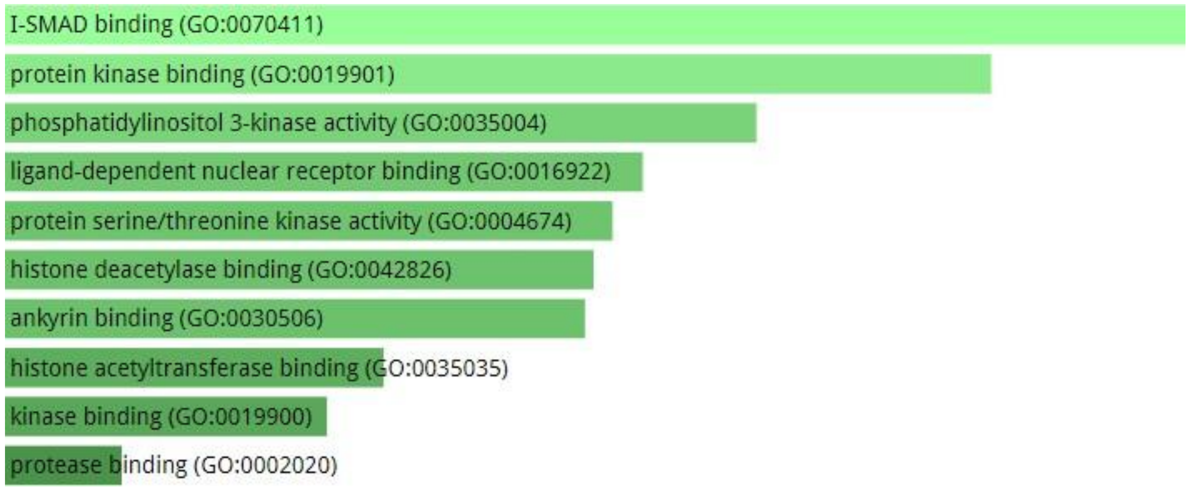

Specific + transversally IGC genes (24 genes)

GO Biological Process 2018

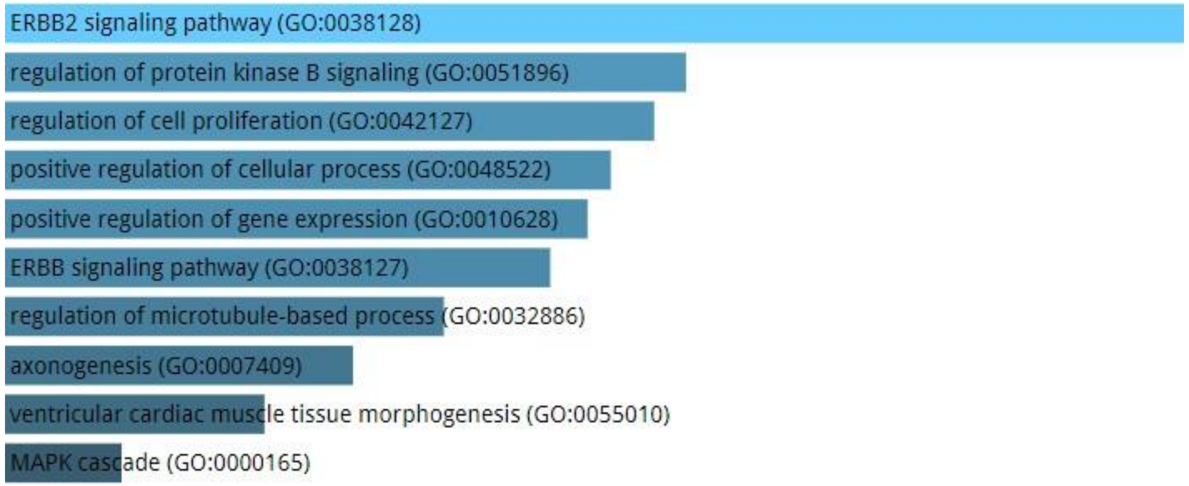

GO Molecular Function 2018

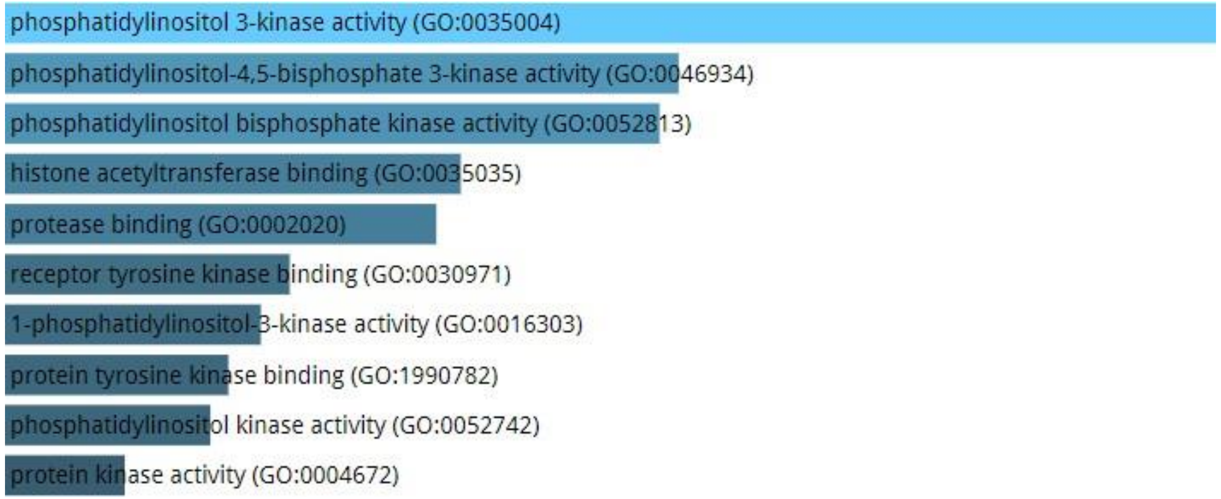

Transversally + Enriched + Specific PCC-NOS genes (25 genes)

GO Biological Process 2018

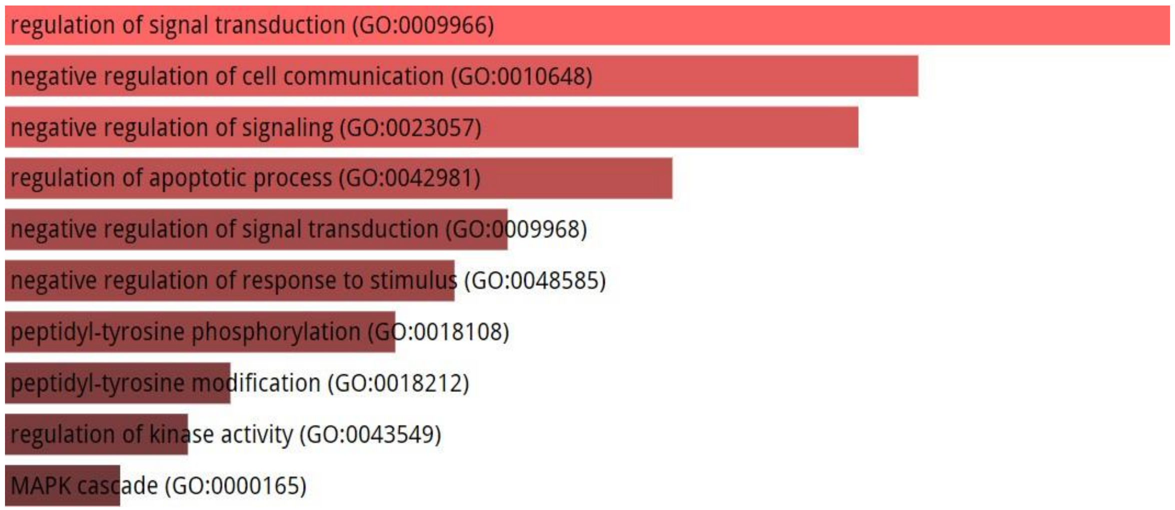

GO Molecular Function 2018

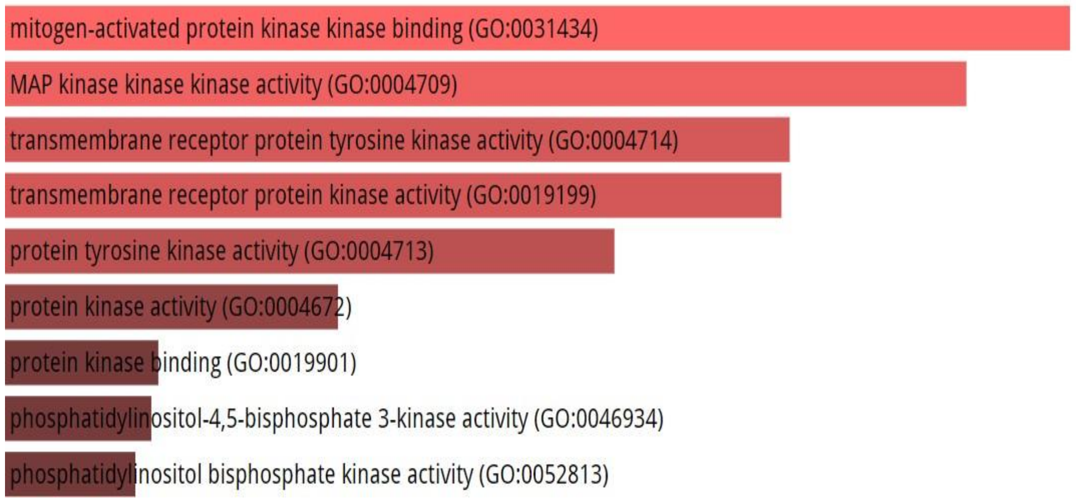

Enriched + Specific PCC-NOS genes (13 genes)

GO Biological Process 2018

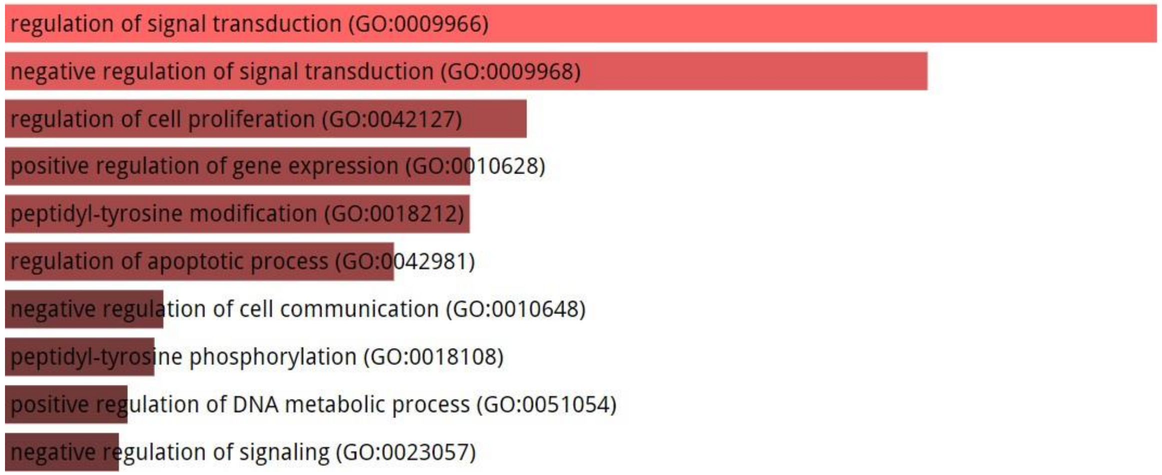

GO Molecular Function 2018

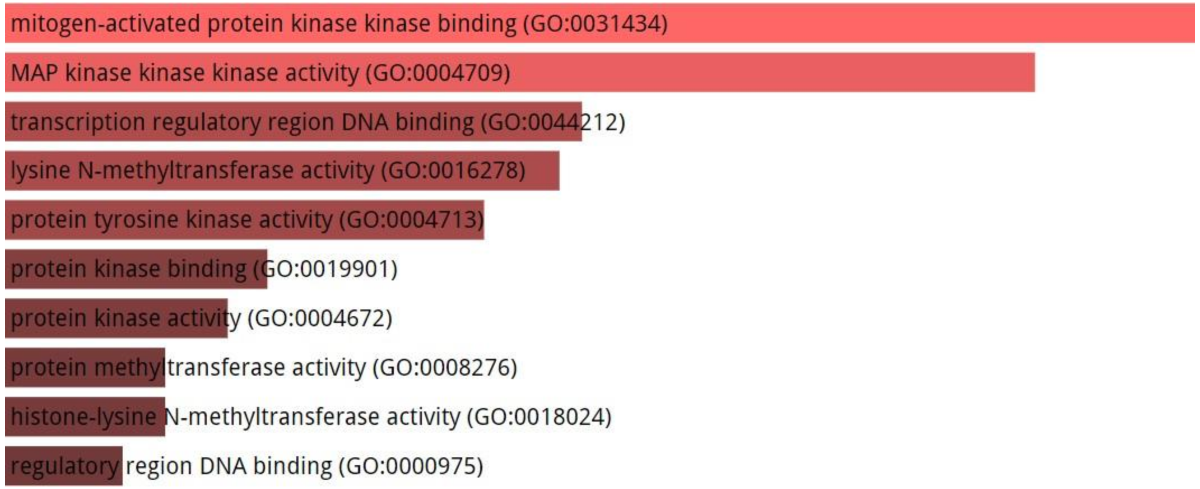

Transversally + Enriched + Specific SRCC genes (27 genes)

GO Biological Process 2018

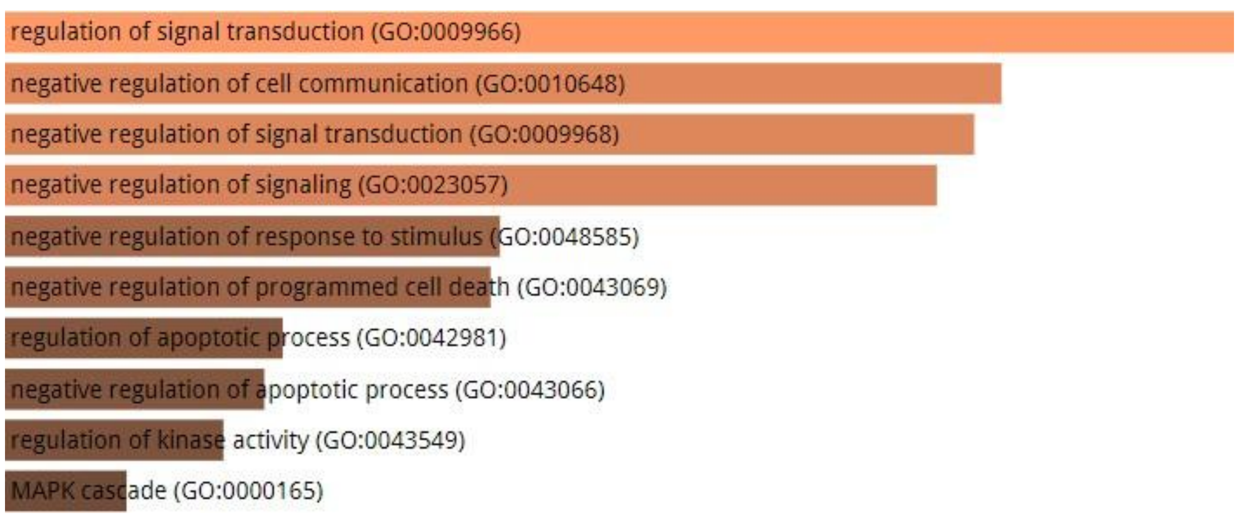

GO Molecular Function 2018

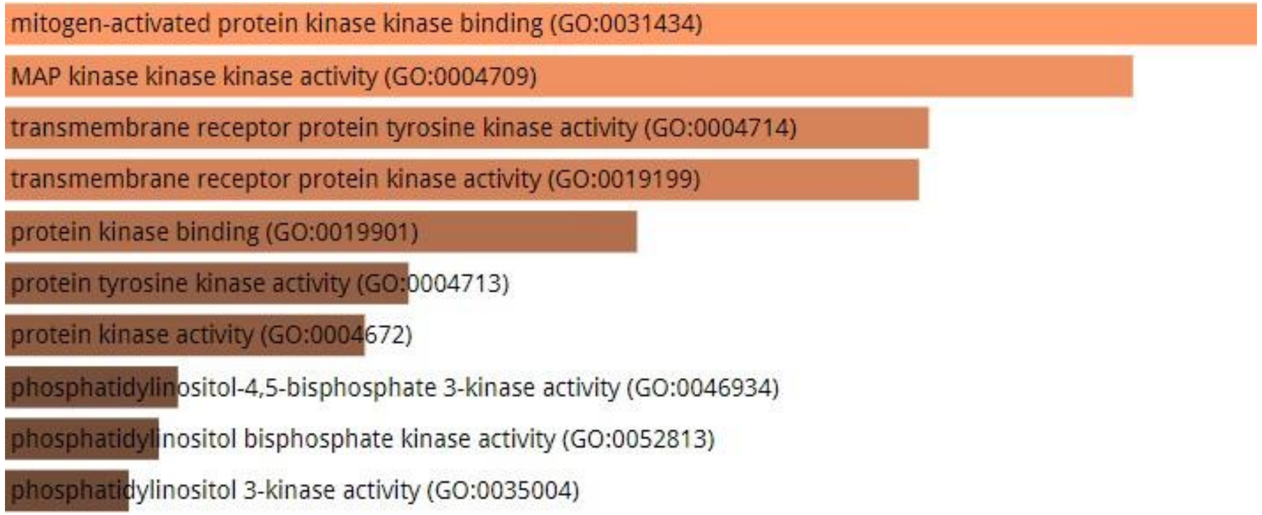

Enriched + Specific SRCC genes (7 genes)

GO Biological Process 2018

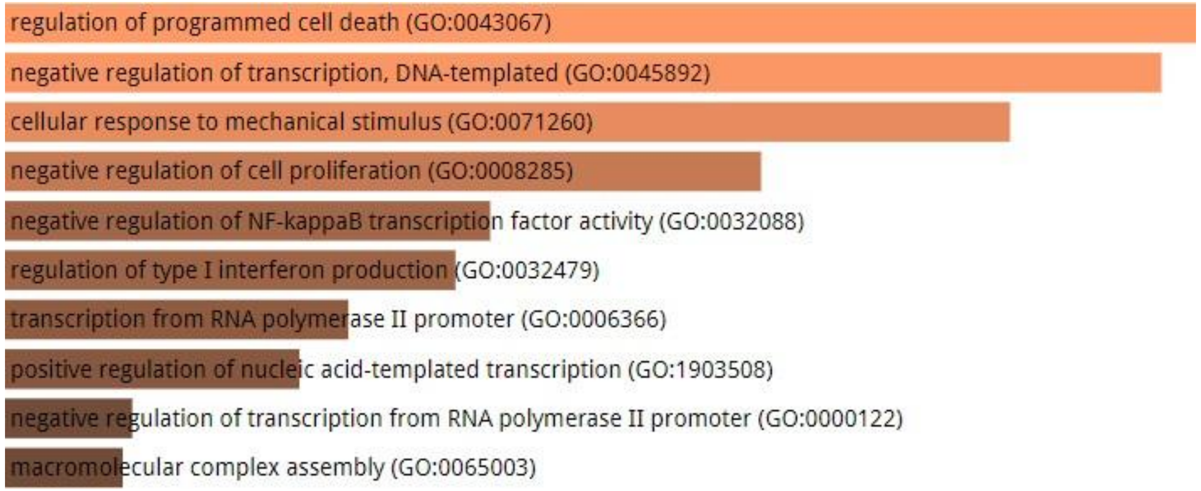

GO Molecular Function 2018

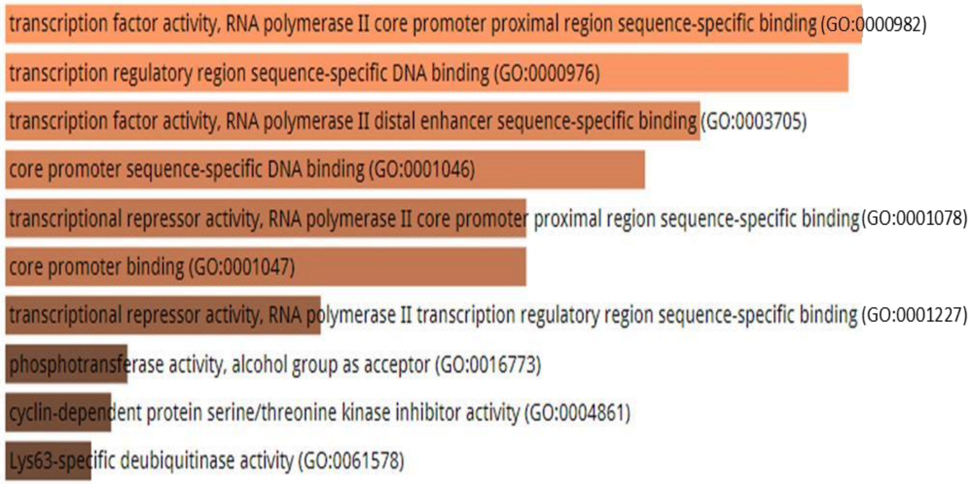

Specific PCC-NOS genes (5 genes)

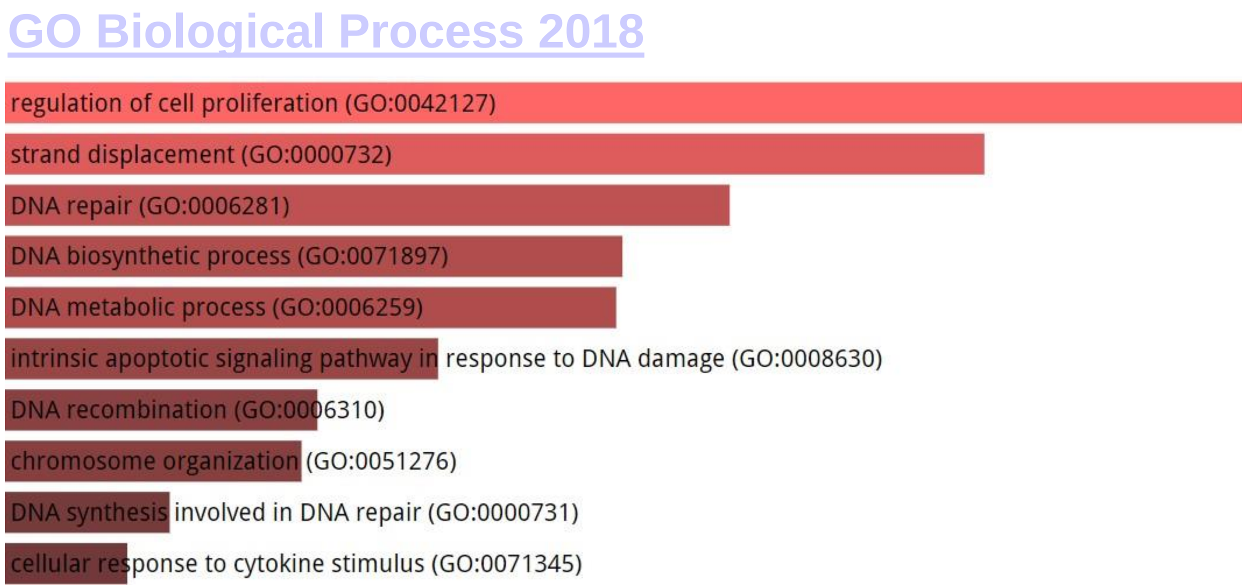

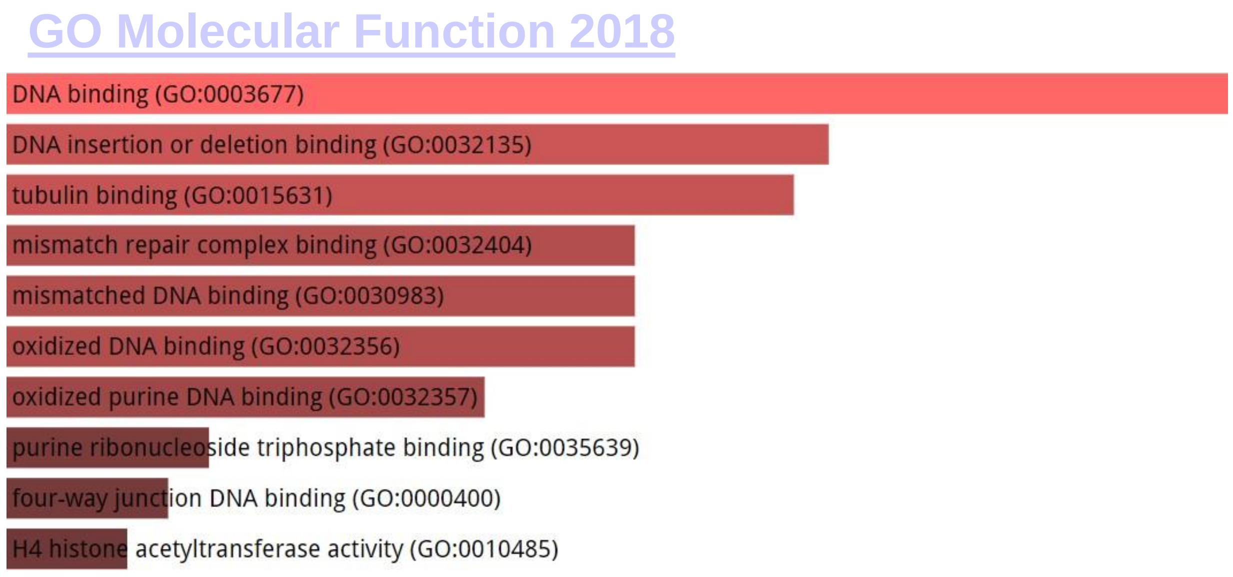

Specific SRCC genes (6 genes)

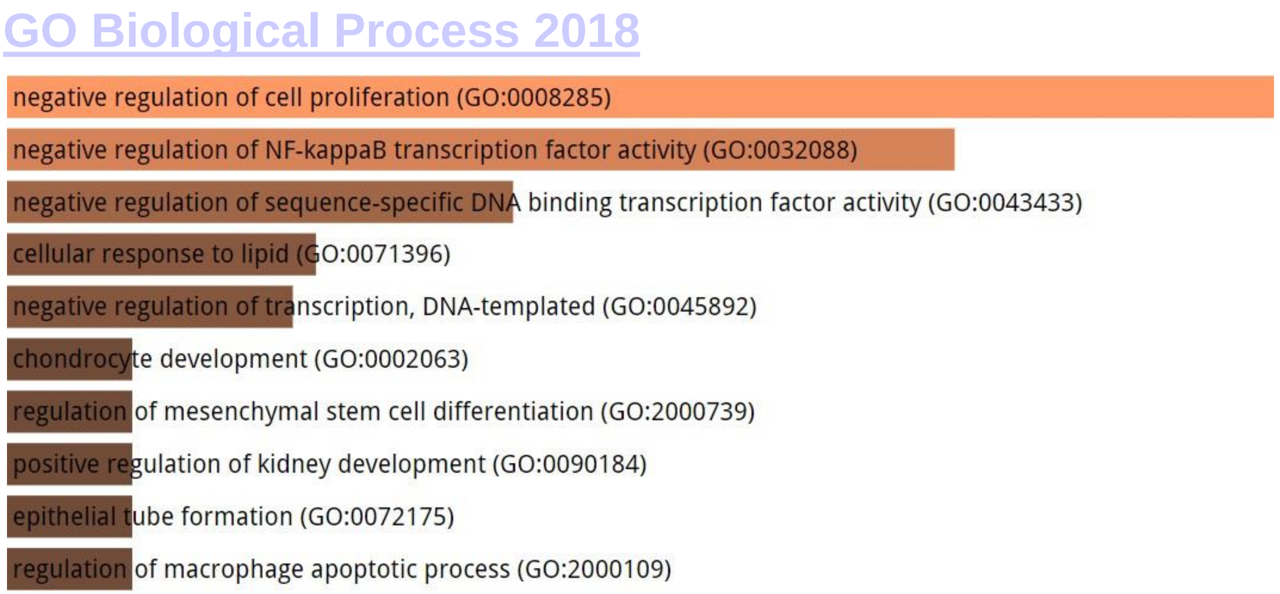

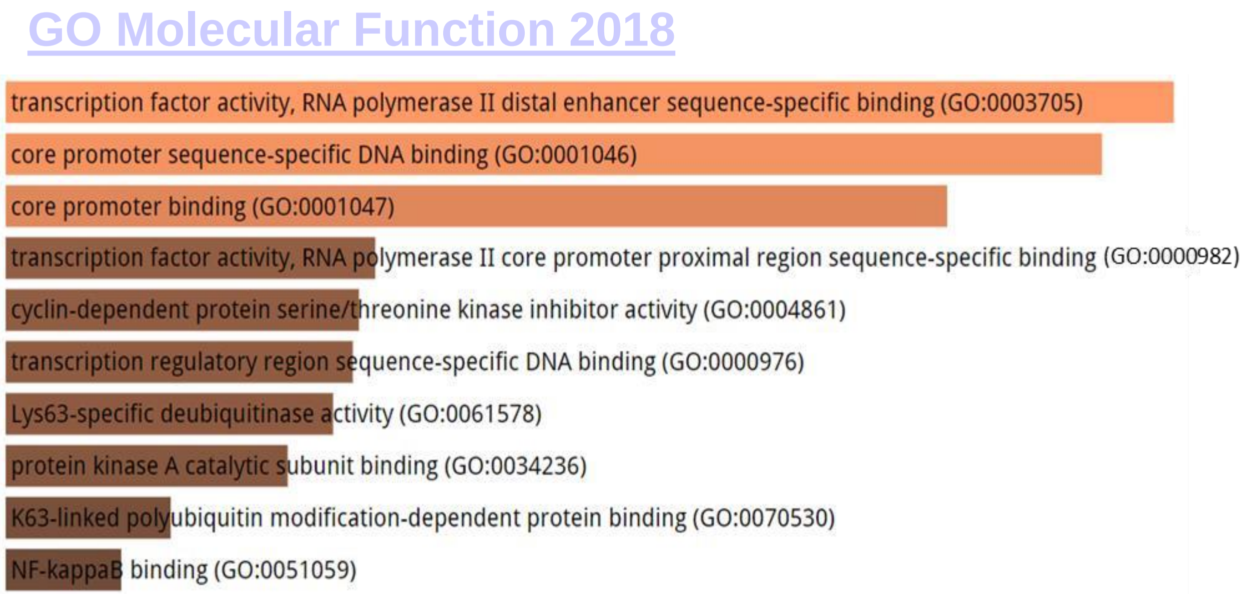

Supplement: Supplementary file 2 — Data S2. GO TERMS, Biological Processes and Molecular Functions retrieved by Enrichr for each genes list: Transversally + Specific DGC/IGC, Specific DGC/IGC genes, Transversally + Enriched + Specific SRCC genes, Specific SRCC genes, Transversally + Enriched + Specific PCC‐NOS genes, and Specific PCC‐NOS genes. [file MOL2-15-2841-s003.pdf]
