# Supplementary material for: Histological and mutational profile of diffuse gastric cancer: current knowledge and future challenges
Source: Mol Oncol. 2021 May 2;15(11):2841–67. doi: 10.1002/1878-0261.12948 (PMC8564639; doi:10.1002/1878-0261.12948)
Supplement: Supplementary file 3 — Table S1. Sample size and details of sequencing methodologies used in the selected publications for analysis. [file MOL2-15-2841-s001.pdf]

Table S1. Sample size and details of sequencing methodologies used in the selected publications for analysis.

Reference/ Sample size	PMID and DOI	Country	Selected Cohort Characteristics	Sequencing Methodologies	Number of genes analysed	Minimum Coverage	Cbio-Portal Identification
C. Kwon *et al.*, Histopathol 2018 n=91	PMID: 28873240; DOI:10.1111/his.13383	Korea	32 SRCC and 59 PCC-NOS cases.	Targeted sequencing (Ion P1 sequencing kit)	77	500 reads	NA
R. Wang *et al.*, Gut 2020 n=43	PMID-31171626; DOI:10.1136/gutjnl-2018-318070	Houston, USA	11 SRCC and 10 PCC-NOS cases. All with peritoneal carcinomatosis.	WES 76 bp paired end technology on Illumina HiSeq4000 platform	NA	7x	NA
K. Chen *et al.* , PNAS 2015 n=78	PMID-25583476; DOI:10.1073/pnas.1422640112	China (North)	78 patients untreated with chemotherapy or radiotherapy prior to stomach surgical resection (WES) and 216 additional patients (targeted re-sequencing). DGC n=155 and IGC n=139	WES 90 bp paired-end technology on Illumina HiSeq2000 platform.	NA (WES)	167x	TMUCIH, PNAS 2015
				Targeted sequecing on Ion Torrent PGM platform.	103	NA	NA
Y. Guo *et al* ., Nature Com 2015 n=147	PMID-29670109; DOI:10.1038/s41467-018-03828-2	Several origins	DGC n=34 and IGC n=75	Distinct WGS approaches from different studies	NA (WGS)	NA	OncoSG, 2018
The Cancer Genome Atlas (TCGA) project n=295	PMID: 25079317; DOI:10.1038/nature13480	Several origins	DGC= 72 not treated with chemotherapy or radiotherapy prior to stomach surgical resection	WES 100bp paired end technology on Illumina HiSeq2000Platform	NA (WES)	60×	TCGA Firehouse Legacy
M. Kakiuchi *et al* ., Nature Gen 2014 n=138	PMID: 24816255; DOI:10.1038/ng.2984	Tokyo, Japan	WES of 30 DGC patients that exhibited poorly differentiated cancer cells and/or signet ring cells with prominent scirrhous stroma in histological analysis. DGC n=87 and IGC n=51	WES 100bp paired end technology on Illumina HiSeq 2000 Platform	NA (WES)	NA	Univ.Tokyo Nat. Gen. 2014
				Targeted sequencing using 150-bp paired-end reads on Illumin HiSeq 2500 platform.	46	20x	NA
K. Wang *et al.* , Nature Gen 2014 n=100	PMID: 24816253; DOI:10.1038/ng.2983	Hong Kong	Matched gastric cancer pairs of tumor and normal. IGC n=18	WGS on 100 bp paired-end technology on Illumina HiSeq2000 platform. DNA SNP array genotyping analysis was performed in parallel for the whole set of samples.	NA (WGS)	84x	(Pfizer and UHK, Nat Genet 2014)
H. Cai et al., J Transl Med 2019 n=153	PMID-31164161; DOI:10.1186/s12967-019-1941-0	China	Cohort with 25 stage I, 39 stage II, and 69 stage III gastric cancers. DGC n= 50 and IGC n=47	Targeted sequencing-Illumina Next 500 platform	612	594 ×	-
K. Wang et al., Nature Gen 2011 n=22	PMID-22037554; DOI:10.1038/ng.982	Hong Kong	DGC n=29 and IGC n=41	WES-Illumina Genome Analyzer II× or HiSeq 2000 platforms	NA (WES)	116×	UHK Nat.Gent. 2011
J.H. Choi *et al* ., Exp & Mol Med 2018 n=23	PMID-29622765; DOI:10.1038/s12276-017-0009-6	Korea	23 DGC patients undergoing gastrectomy	WES-101 bp paired-end reads using Illumina GAIIx	NA (WES)	126x	-

Footnote: NA, not available; WES: Whole Exome Sequencing; WGS: Whole Genome Sequencing
