# Supplementary material for: Histological and mutational profile of diffuse gastric cancer: current knowledge and future challenges
Source: Mol Oncol. 2021 May 2;15(11):2841–67. doi: 10.1002/1878-0261.12948 (PMC8564639; doi:10.1002/1878-0261.12948)
Supplement: Supplementary file 4 — Table S2. Details of Clinical trial registered at ClinicalTrials.gov directed to gastric cancer and often including DGC patients. [file MOL2-15-2841-s002.pdf]

Table S2. Details of Clinical trials registered at ClinicalTrials.gov directed to gastric cancer and often including DGC patients.

ClinicalTrials. gov Identifier	Title of the clinical trial	Strategy	Potencial molecular targets beyond 5-FU and cisplatin treatment	Status
NCT03977220	Nab-paclitaxel combined with S-1 treating diffuse type of stage Ⅲ gastric cancer as adjuvant setting (NORDICA)	Paclitaxel (microtubules stabilization) and S-1	Microtubules	Not yet recruiting
NCT04254107	A Safety Study of SGN-TGT in Patients With Advanced Cancer (SEA-TGT)	Blocking of PD-1 receptor	PD-1 receptor	Recruiting
NCT01717924	Evaluation of Surgery Versus Primary Chemotherapy in Resectable Signet Ring Cell Gastric Adenocarcinoma (ADCI002/PRODIGE) (Phase II/III)	Comparing primary surgery versus primary chemotherapy followed by surgery. Epirubicin for topoisomerase II inhibition; Cisplatin; 5-fluorouracil	Topoisomerase II	Recruiting
NCT01260701	Akt Inhibitor MK2206 in Treating Patients With Advanced Gastric or Gastroesophageal Junction Cancer	Study of Akt inhibitor function (MK2206). Inhibition of enzymes needed for cell growth such as Serine/Threonine protein kinase Akt (protein kinase B).	Protein kinase B (PKB)	Completed
NCT00103324	S0413 Lapatinib in Treating Patients With Locally Advanced or Metastatic Stomach Cancer	Inhibition of enzymes needed for cell growth such as Erk1/2 and Akt phosphorylation.	Erk1/2 & Akt phosphorilation	Completed
NCT00991952	Irinotecan Hydrochloride With or Without Alvocidib in Treating Patients With Advanced Stomach or Gastroesophageal Junction Cancer That Cannot Be Removed By Surgery	Use of Hydrochloride for Inhibition of Topoisomerase I; Alvocidib for inhibition of phosphorylation of cyclin-dependent kinases (CDKs) and down-regulation of cyclin D1 and D3 expression	Topoisomerase I CDKs phosphorilation & Cyclin D1 and D3	Completed
NCT00084604	Irinotecan, Cisplatin, and Bevacizumab in Treating Patients With Unresectable or Metastatic Gastric or Gastroesophageal Junction Adenocarcinoma	Combined use of Irinotecan and Cisplatin together with Bevacizumab. Irinotecan act as a Topoisomerase I inhibitor; Bevacizumab: Binds to VEGF and prevent binding to VEGF receptor	Topoisomerase I and VEGF receptors	Completed
NCT00061932	Bortezomib With or Without Irinotecan in Treating Patients With Cancer of the Gastroesophageal Junction or Stomach	Studying the effect of bortezomib with or without irinotecan. Bortezomib: ubiquitin-proteasome pathway; Irinotecan: Topoisomerase I inhibitor	Ubiquitin-proteasome pathway; Topoisomerase I	Completed
NCT01285557	Diffuse Gastric and Esophagogastric Junction Cancer S-1 Trial (DIGEST)	Evaluate the safety and efficacy of S-1 and Cisplatin compared to 5-FU and Cisplatin. Inhibition of dihydro-pyrimidine dehydrogenase	Conventional chemotherapy targets	Completed
NCT01576380	A Phase II Study to Evaluate Efficacy and Safety of Dovitinib (TKI258) in Advanced Scirrhous Gastric Carcinoma Patients	Inhibition of angiokinase (phosphorylation of type III-V receptor tyrosine kinase - RTKs)	angiokinase inhibition	Completed
NCT00879333	Safety and Efficacy of RAD001 (Everolimus) Monotherapy Plus Best Supportive Care in Patients With Advanced Gastric Cancer (AGC) (GRANITE-1)	Inhibition mTOR	mTOR	Completed
NCT01197885	Efficacy and Safety Study of Multiple Doses of IMAB362 in Patients With Advanced Gastroesophageal Cancer (MONO)	IMAB362 used as monoclonal antibody against CLDN18.2	CLDN18.2	Completed
NCT01457846	Efficacy and Safety of AZD4547 Versus Paclitaxel in Patients With Advanced Gastric or Gastro-oesophageal Cancer (SHINE)	Inhibition of ATP-competitive RTK (FGFR2 inhibitor)	FGFR2	Completed+B 4:F17

Footnote: 5-FU: 5-fluorouracil; PD-1: Programmed cell death protein 1; Akt: Protein Kinase B; Erk: extracellular signal-regulated kinase; CDK: Cyclin dependent kinase; VEGF: Vascular endothelial growth factor; AGC: advanced gastric cancer; mTOR: mammalian target of rapamycin; CLDN: Claudin; ATP: Adenosine triphosphate; RTK: Tyrosin kinase receptor; FGFR2: Fibroblast Growth Factor Receptor 2; Legend: Green was used to highlight clinical trials that specifically mention DGC; Red was used to highlight clinical trials that specifically mention SRCC.
